# Sm^3+^-Doped Y_2_Ge_2_O_7_ Orange-Red Phosphor Prepared via the Sol–Gel Method for Multimodal Applications: A Study of of the Effect of Dopant Concentration on Structural and Optical Properties

**DOI:** 10.1021/acsomega.6c05178

**Published:** 2026-10-13

**Authors:** Amanda Alencar Arrais de Garcia, Isabella Lima Caetano, Francisco Alisson da Silva Torquato, Marcelo Marques, Lara K. Teles, Renato Borges Pontes, Lauro June Queiroz Maia, Ricardo Costa de Santana

**Affiliations:** † Instituto de Física, 67824 Universidade Federal de Goiás, Goiânia, Goiás 74690-900, Brazil; ‡ 74360 Instituto Tecnológico de Aeronáutica, São José dos Campos, São Paulo 12228-900, Brazil

## Abstract

This work reports, for the first time, the synthesis of single-phase trivalent samarium-doped yttrium pyrogermanate (Y_2_Ge_2_O_7_:*x*Sm^3+^) phosphors via the sol–gel method. The crystalline structure, photoluminescence mechanisms, energy transfer dynamics, and optical thermometry properties were systematically investigated. The experimental optical bandgap (∼5.99–6.10 eV) shows good agreement with those obtained from the density functional theory (DFT) calculations. Upon Sm^3+^ incorporation, a concentration quenching effect reduces the quantum yield from 8.1% to 0.3%, correlated with a reduction in the average lifetime from 2.08 to 0.61 ms. Analysis of the decay kinetics via the Van Uitert model indicates a dipole–dipole interaction governing the energy transfer, achieving a cross-relaxation efficiency of ∼73% at 4.0 mol %. Remarkably, the CIE coordinates, correlated color temperature (CCT), and color purity (CP) remain independent of the dopant content, exhibiting an exceptional red color purity above 95% for all samples. These results demonstrate that Y_2_Ge_2_O_7_ *x*Sm^3+^ phosphors are promising candidates for solid-state lighting and multifunctional optical applications. Additionally, the ratio between emission sub-bands from ^4^ *G*_5/2_ → ^6^ *H*_9/2_ to ^4^ *G*_5/2_ → ^6^ *H*_5/2_ provides a relative sensitivity value (*S*_R_) for the sample with 4.0 mol % up to 0.23%/K, indicating a potential use as an optical sensing thermometer.

## Graphical abstract

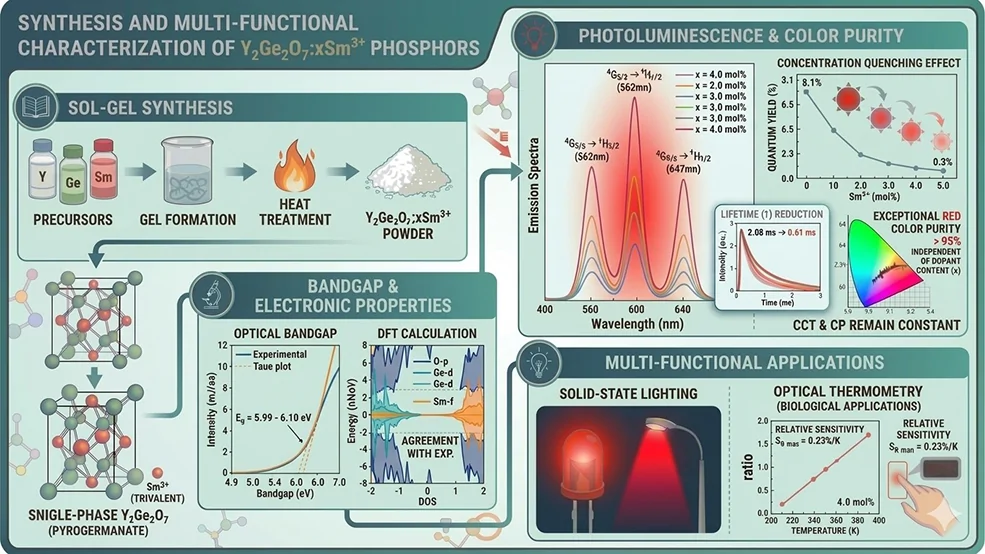

## 1 Introduction

The Sm^3+^-doped germanate crystals have been explored due to their well-known green, orange, and red emissions coming from the electronic transitions ^4^ *G*_5/2_ → ^6^ *H*_5/2,7/2,9/2,11/2_, which could be applied in many photonics devices, such as solid-state lasers, optical amplifiers, high-density optical data storage, radiation detection, and others,^1–3^ even with lower emission quantum yields due to nonradiative decay. Particularly due to the strong red emission around 600 nm (^4^ *G*_5/2_ → ^6^ *H*_7/2_) of Sm^3+^, Sm-doped phosphors are excellent candidates for integration with commercially available blue semiconductor chips, that is, these Sm-doped phosphors effectively compensate for the deficiency in the red spectral region typically observed in conventional LEDs.^1^

Beyond the lighting devices, investigation into the relative contribution between green (^4^ *G*_5/2_ → ^6^ *H*_5/2_) and red (^4^ *G*_5/2_ → ^6^ *H*_9/2_) emissions of Sm^3+^, called the **R/G** ratio, has gained attention in recent years due to the possibility that a higher asymmetry site improves the red component and positively impacts the color rendering index when applied to red-light-emitting devices.^4,5^ The emission enhancement can be explained by the magnetic character of the ^4^ *G*_5/2_ → ^6^ *H*_5/2_ transition, whose probability and intensity remain insensitive to the surrounding crystallographic environment, whereas the ^4^ *G*_5/2_ → ^6^ *H*_7/2_ and ^4^ *G*_5/2_ → ^6^ *H*_9/2_ ones have a dipole electric contribution and are strongly sensitive to the symmetry site.^6^ Therefore, the obtention of germanate phases with a higher asymmetry at the Sm^3+^ site is necessary to enhance the orange-red emissions and improve the performance as a red emitter.

On the other hand, over a broad temperature range, the ^4^ *G*_5/2_ → ^6^ *H*_J_ transitions of Sm^3+^ ions are also commonly used as thermometric parameters, particularly due to the temperature-dependent changes in the red-to-green (**R/G**) emission components, mainly focusing on the ^4^ *G*_5/2_ → ^6^ *H*_9/2_/^4^ *G*_5/2_ → ^6^ *H*_5/2_ emission ratio.^7^ For instance, in SrMoO_4_:Sm^3+^, the **R/G** ratio rises with temperature, reaching a relative sensitivity of 0.21% K^–1^ at 458 K.^8^ Similarly, in the double perovskite Ca_2_LaNbO_6_:Sm^3+^, the **G/R** ratio also increases with temperature, exhibiting a sensitivity of 0.2% K^–1^ near room temperature.^9^ Furthermore, Long et al.^10^ demonstrated that the **R/G** ratio remains a reliable metric for temperature readout in Y_2_MgTiO_6_:Sm^3+^, showing no signs of saturation in the luminescence intensity ratio (LIR) up to 500 K. These findings highlight the robustness of the **R/G** ratio as a ratiometric parameter for optical thermometry in Sm^3+^-doped phosphors.

Among germanate hosts, Sm^3+^ codoped Y_2_Ge_2_O_7_:Pr^3+^ has recently attracted attention due to its ability to modulate electron traps in this system.^11^ However, there is currently no reported information on the nonradiative depopulation mechanism of the ^4^ *G*_5/2_ level in this phase, particularly regarding the quantification of cross-relaxation rates. Moreover, optical thermometry studies are also lacking in the Y_2_Ge_2_O_7_:Sm^3+^ samples. We find that previous studies involving other lanthanide dopants have explored both thermally coupled^12^ and nonthermally coupled methodologies^13^ in Y_2_Ge_2_O_7_. In the context, the first report about the use of the asymmetric ratio as a thermometric parameter in the Y_2_Ge_2_O_7_:Eu^3+^ phase^14^ showed an increase in the ^5^D_0_ →^7^F_2_/^5^D_0_ →^7^F_1_ emission ratio between 20 and 55 °C, indicating that rising temperature leads to a reduction in site symmetry within the YO_7_ geometry. However, Y_2_Ge_2_O_7_:Eu^3+^ exhibits minimal concentration quenching, which limits the investigation into how cross-relaxation mechanisms influence the asymmetric ratio that could be used in thermometric applications.

This article explores the PL quenching mechanisms and thermometric properties of Y_2_Ge_2_O_7_ samples synthesized with different Sm^3+^ concentrations. Based on lifetime measurements and luminescence analysis, the dipole–dipole interaction was found to dominate the cross-relaxation mechanisms, with an efficiency of nearly 73% observed in samples doped with 4 mol % Sm^3+^. Additionally, we found the thermal relative sensitivity (*S*_R_) of approximately 0.23%.K^–1^ derived from the increase in the asymmetric **R/G** ratio using sub-Stark level emissions as temperature rises. The small variation in the contribution of the total integrated area intensity from the green component, in comparison to the orange and red components, also supports the lower correlated color temperature (CCT) as the samples are heated, indicating that Y_2_Ge_2_O_7_:Sm^3+^ phosphor is a promising candidate to produce red light for LED devices or temperature sensing.

## 2 Materials and Methods

### 2.1 Synthesis

A series of six powder samples with nominal compositions of 0.2 mol % (Y_1.992_Sm_0.008_Ge_2_O_7_), 0.5 mol % (Y_1.980_Sm_0.020_Ge_2_O_7_), 1.0 mol % (Y_1.960_Sm_0.040_Ge_2_O_7_), 2.0 mol % (Y_1.920_Sm_0.080_Ge_2_O_7_), 3.0 mol % (Y_1.880_Sm_0.120_Ge_2_O_7_), and 4.0 mol % (Y_1.840_Sm_0.160_Ge_2_O_7_) were synthesized by a sol–gel route following the procedure described in previous works.^13,14^ The molar fraction mol % substituted *x* is determined using the total metal ions in the host; in this case mol % = *x*/4. All chemical reagents and solvents, including citric acid monohydrate (C_6_H_8_O_7_.H_2_O, Sigma-Aldrich, 99.91% purity), ethanol (C_2_H_5_OH, Exodo Científica, 99.5% purity), samarium nitrate hexahydrate Sm­(NO_3_)_3_·6H_2_O, Sigma-Aldrich, 99.9% purity), yttrium nitrate hexahydrate (Y­(NO_3_)_3_·6H_2_O, Sigma-Aldrich, 99.8% purity), germanium oxide (GeO_2_, Sigma-Aldrich, 99.998% purity), and tetramethylammonium hydroxide solution (TMAH, C_4_H_13_NO, Sigma-Aldrich 25% volume in methanol), were used without further purification. The reagent amounts used in the synthesis are given in Table S1.

A sol–gel route was used to form the polymeric precursor solution, where the citric acid monohydrate was diluted in ethanol under vigorous stirring for 30 min. At the same time and separately, the Y­(NO_3_)_3_·6H_2_O was diluted in ethanol and ultrapure water in a volumetric ratio of 3:1 ethanol/water; the Sm­(NO_3_)_3_·6H_2_O was diluted in ethanol, and the germanium dioxide was reacted with TMAH, C_4_H_13_NO with ultrapure water, with a ratio of 0.24 g GeO_2_: 2.0 mL TMAH: 1.5 mL H_2_O. Afterward, the nitrate solutions were mixed under vigorous stirring for 30 min and then left to rest at room temperature for 24 h, producing stable, transparent sols. The sol was dried in an oven at 160 ◦C for 24 h. After the material was crushed in an agate mortar to obtain fine powder, it was followed by heat treatment at 200 °C for 48 h. After grinding again, the powders were calcined at 400 °C for 24 h in a muffle furnace under an ambient atmosphere. This was followed by grinding to form fine powders in an agate mortar. Finally, the powders were heat treated at 1100 °C for 1 h with a heating rate of 10 °C/min.^13,14^ Figure 1 displays a schematic illustration of the preparation of Y_2_Ge_2_O_7_:*x*Sm^3+^ phosphors.

**Figure 1:**
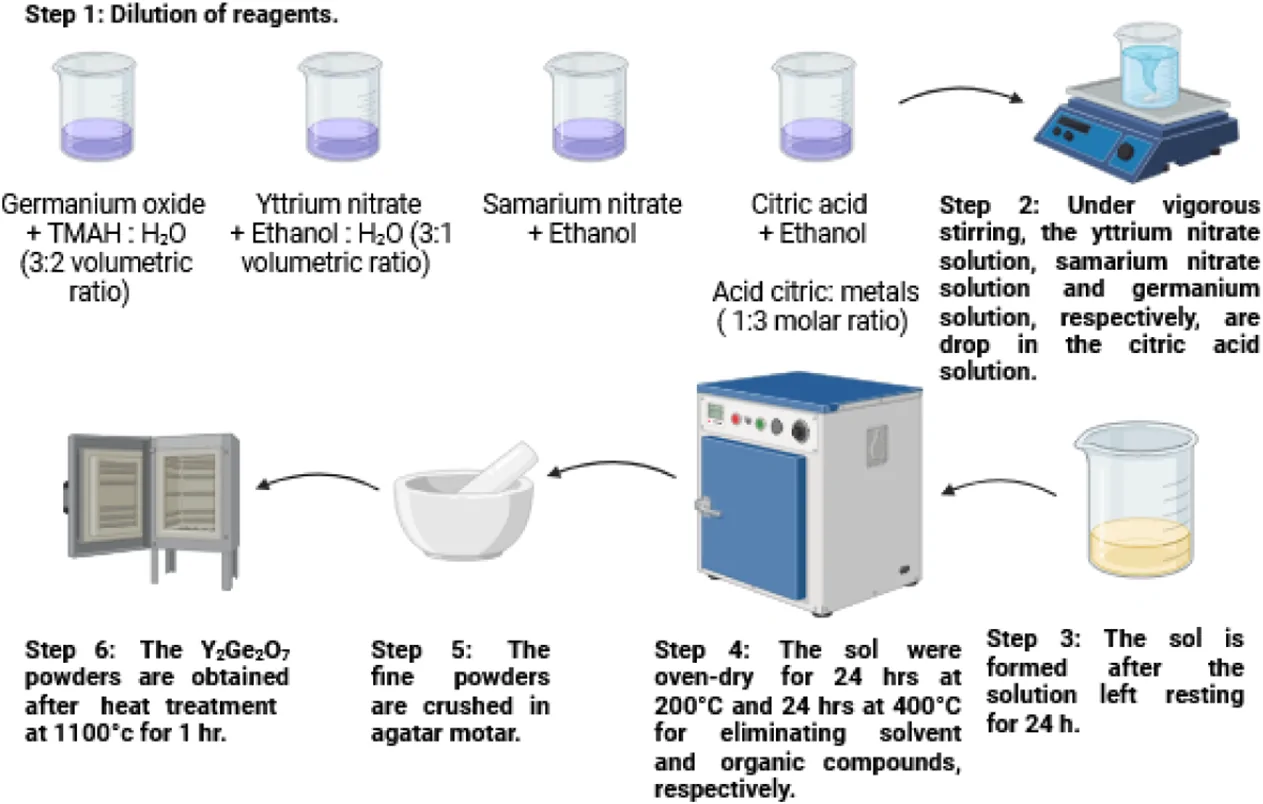
Schematic flow diagram of the synthesis method used.

### 2.2 Computational Details

To simulate the experimental doping of Y_2_Ge_2_O_7_:*x*Sm^3+^, a 176-atom supercell containing 32 Y sites (16 formula units of Y_2_Ge_2_O_7_) was employed. The substitution of a single Y^3+^ site with a Sm^3+^ ion corresponds to a dopant concentration of *x* = 3.125 mol%, which directly reflects the intermediate-to-high experimental doping regime (0.2–4.0 mol%). Furthermore, a two-atom substitution model (*x* = 6.250 mol%) was investigated to represent a high-concentration regime. All calculations were performed within the framework of density functional theory (DFT) as implemented in the Vienna Ab Initio Simulation Package (VASP).^15,16^ The exchange-correlation effects were treated using the Perdew–Burke–Ernzerhof (PBE) generalized gradient approximation.^17^ The interaction between core and valence electrons was described using the projector augmented wave (PAW) method.^18,19^ The valence configurations considered were: 4*s* ^2^4*p* ^6^4*d* ^1^5*s* ^2^ for Y (*Z*_val_ = 11), 4*s* ^2^4*p* ^2^ for Ge (*Z*_val_ = 4), 2*s* ^2^2*p* ^4^ for O (*Z*_val_ = 6), and 5*s* ^2^5*p* ^6^5*d* ^1^6*s* ^2^ along with additional pseudovalence states for Sm (*Z*_val_ = 16, utilizing a [Xe]­4*f* ^5^ frozen core). All calculations were spin-polarized to account for the local magnetic configurations.

A plane-wave basis set with a kinetic energy cutoff of 550 eV was employed. Structural relaxations, including both lattice parameters and atomic positions, were carried out until the residual forces were below 10^–2^ eV/Å, with an energy convergence criterion of 10^–6^ eV. Brillouin zone integration was performed using an automatically generated Γ-centered *k*-mesh of 6 × 6 × 6 for structural optimization and a denser 11 × 11 × 11 grid for electronic properties, the latter employing the tetrahedron method with Blöchl corrections.^20^ Spin–orbit coupling (SOC) was neglected due to the large system size and the fact that the primary host band edge states (O 2*p*, Ge 4*p*, Y 4*d*) are not dominated by heavy relativistic splitting, while the Sm^3+^4*f* shell is localized within the core.

To provide band gap values faithful to experimental results, we employ the DFT-1/2 quasiparticle correction of the electronic structure.^21,22^ This approach provides bandgap accuracy comparable to computationally intensive methods, such as hybrid functionals or the GW approximation, while maintaining the efficiency of standard DFT. The DFT-1/2 method, based on Slater’s half-occupation scheme,^23^ modifies the Kohn–Sham potential by subtracting a self-energy potential derived from the difference between neutral and half-ionized atomic potentials. To prevent the unphysical overlap of self-energy Coulomb tails with neighboring sites, the potential is truncated by a step function. The cutoff radius was determined variationally by maximizing the bandgap, a procedure sensitive to the atomic orbitals defining the valence band maximum (VBM). An optimized cutoff parameter of CUT = 2.7 a.u. was applied to the oxygen ions.

This methodology has been successfully validated across diverse systems, including halide and double perovskites.^24–28^ Parameters for the DFT-1/2 functionals were optimized using the minushalf code.^29^ Data postprocessing was conducted via VASPKIT,^30^ and the electronic density of states (DOS) was analyzed and rendered using the sumo toolkit.^31^

### 2.3 Characterization

The powders of Y_2_Ge_2_O_7_:*x*Sm^3+^ were investigated by X-ray powder diffraction (XRPD) using a Bruker D8 Discover diffractometer using Cu*K*_α1_ radiation in the 2θ range from 3° to 100°, with a step of 0.01°/min, at CRTi (Regional Centre for Technological and Innovation Development). The morphology and elemental mapping were obtained using a Jeol JSM-6610 scanning electron microscope (SEM) equipped with an EDS (Energy Dispersive X-ray Spectroscopy) Thermo Scientific NSS Spectral Imaging from Japan, and High-Resolution Transmission Electron Microscopy (HRTEM) and Selected Area Electron Diffraction (SAED) images were obtained using the JEOL microscope, model JEM 2010, operating at 200 keV, at LabMic (Multiuser High Resolution Microscopy Laboratory, IF-UFG). X-ray Photoelectron Spectroscopic (XPS) measurements were conducted using a Thermo Fischer Scientific NEXSA G2 instrument equipped with an Al K-Alpha X-ray source. All analyses and fits of XPS spectra were made using Thermo Scientific Avantage v6.11.2 software. The diffuse reflectance spectra were collected using a PerkinElmer spectrophotometer, model Lambda WB1050, using a Praying Mantis accessory and barium sulfate powder (Sigma-Aldrich) as a reference. For photoluminescence (PL) experiments (excitation, emission, lifetime, and quantum yield), a Horiba Jobin-Yvon Fluorolog FL3–221 spectrofluorometer with dual monochromators and a Hamamatsu photomultiplier was used. A 450 W Xe lamp was used as an excitation source for PL measurements, with a bandpass of 0.8 nm, an increment of 0.3 nm, and an integration time of 0.2 s. PL decay curves were recorded using an Xe pulse lamp with excitation at 402.7 nm, where the emission at 601 nm was monitored. For quantum yield (QY) experiments, an integrating sphere coupled to a fluorimeter by means of optical fibers, and sample holders made of Spectralon were used. The QY values (internal and external) were calculated by using the equations from Wrighton et al:^32^ iQY (%) = (*E*_s_/(*R*_std_ – *R*_s_)) × 100, and eQY = (*E*_s_/*R*_std_) × 100, where *E*_s_ is the number of emitted photons from the sample, *R*_std_ and *R*_s_ are the number of reflected photons from the reflection standard and the sample, respectively, and (*R*_std_ – *R*_s_) is the number of photons absorbed by the sample. Thermal measurements were performed using an ultrathermostatic bath , model CT-281–9 from Cientec, considering the temperature range from 293 to 332.6 K, where each spectrum was recorded after a 30 min thermal stabilization period, using controlled 5 °C increments between each step.

## 3 Results and Discussion

### 3.1 Structural Characterization

In order to identify the crystalline phase of Y_2_Ge_2_O_7_:*x*Sm^3+^ phosphors, XRPD patterns were collected and illustrated in Figure 2a, with all characteristic lines of the standard compound with a *P*4_3_2_1_2 space group according to COD card number 2016209^33^, in accordance with the report by Barbosa et al.,^14^ revealing that all synthesized samples consisted of a single phase of the tetragonal system, with no occurrence of spurious phases, suggesting that the Sm^3+^ ions are well incorporated into the crystalline lattice. In the host matrix, which possesses a distinct tetragonal crystal structure, this localized “peak inversion” effect (shifting right, then left) is a classic signature of a two-stage structural response. When a larger rare-earth dopant (such as Bi^3+^, Sm^3+^, Tb^3+^, or Pr^3+^) replaces Y^3+^, the initial shift to the right happens due to interstitial placement or vacancies, while the subsequent shift to the left represents true substitutional swelling. A similar result was first observed and discussed by Tiagy et al. for Y_2_Ge_2_O_7_:Eu^3+^,^34^ and later by Mal et al. for Bi^3+^-doped Y_2_Ge_2_O_7_ (Yb–Er),^35^ in terms of the coordination environment, the difference in ionic radii, and the dopant levels.

**Figure 2:**
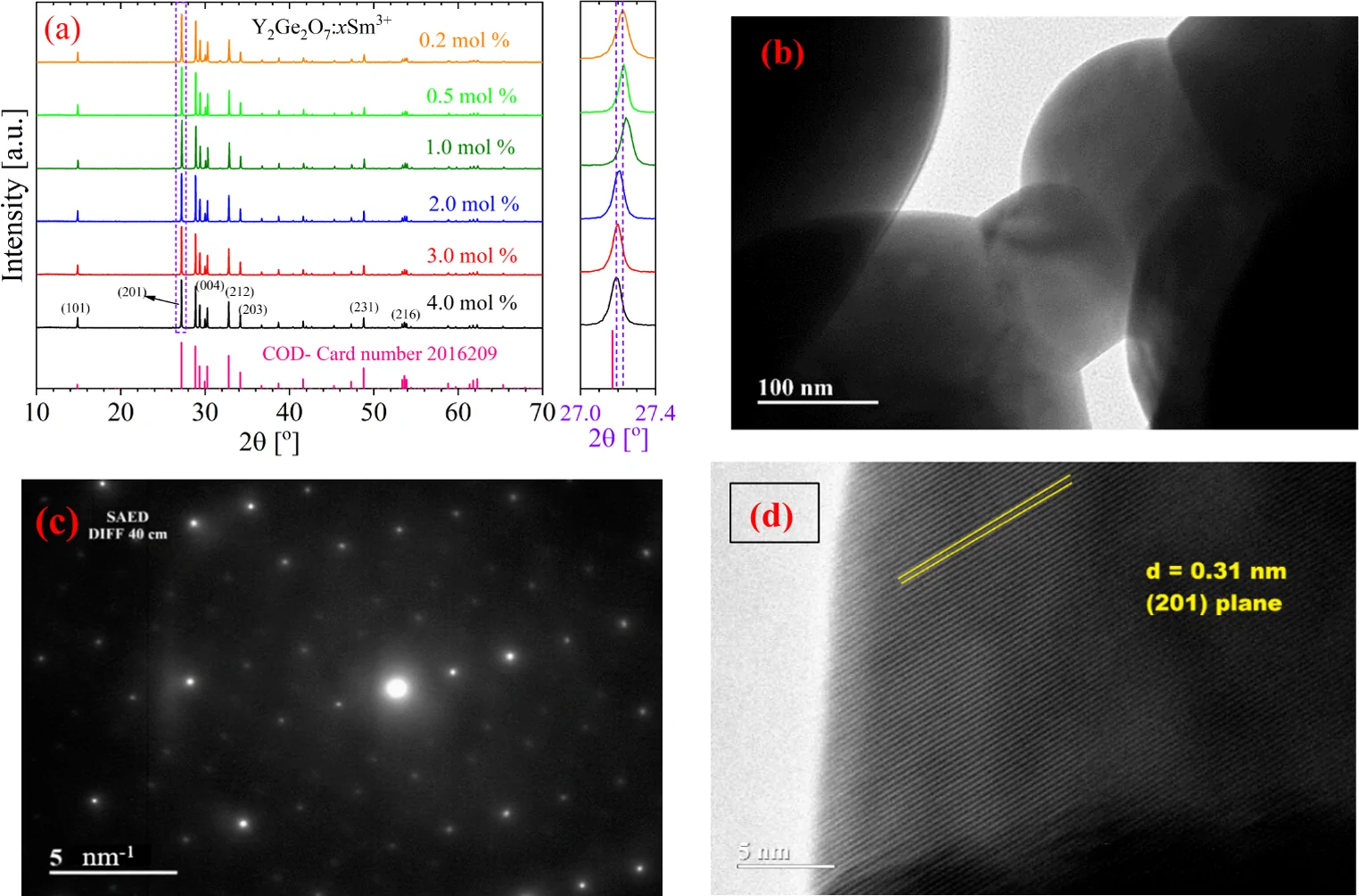
(a) X-ray powder diffraction patterns of Sm^3+^-doped Y_2_Ge_2_O_7_ phosphors, compared with the standard data of Y_2_Ge_2_O_7_ (JPCDS no 2016209). (b) TEM, (c) SAED, and (d) high-resolution TEM images of Y_2_Ge_2_O_7_:Sm^3+^ (0.2 mol %).

As presented in the SEM images in Figure S1, as well as in the representative TEM image of Y_2_Ge_2_O_7_:(0.2 mol %) Sm^3+^ powder, Figure 2b, the prepared samples for a sol–gel route to form particles densely packed due to the coalescence process,^14^ consisting of irregular agglomerates, as observed in other phosphors prepared by the sol–gel process.^36,37^ Moreover, the elemental mapping, also shown in colored Figure S1, reveals a uniform distribution of elements such as Y, Ge, and Sm in the particles. The discrete spots observed in the SAED pattern, presented in Figure 2c, provide a qualitative indication of the single-crystalline nature of the particles, a finding structurally validated by the quantitative XRD data. Moreover, the representative HRTEM image in Figure 2d shows high crystallinity of the obtained phosphors, displaying lattice fringes with an interplanar distance of around 0.31 nm, in agreement with the (201) plane,^33^ suggesting the absence of any secondary phase as indicated by PXRD results.

In order to access more information about the Sm^3+^ incorporation in Y_2_Ge_2_O_7_, X-ray photoelectron spectroscopy (XPS) was carried out for Y_2_Ge_2_O_7_:*x*Sm^3+^ (*x* = 0.5, 2.0, and 4.0 mol %) samples. The survey XPS spectra for these samples are shown in Figure 3a–3c, respectively. The figures clearly show all of the elements in the phosphor as O 1s, Y 3d, Ge 2p, and Sm 3d elements. Adsorption of residual gases on the sample surface introduced spurious carbon and oxygen spectral bands. A quantitative assessment of the surface contamination was precluded by its spatial inhomogeneity and temporal instability. Also, a correction of the energy peaks was done on the basis of the C 1s peak at 284.4 eV.

**Figure 3:**
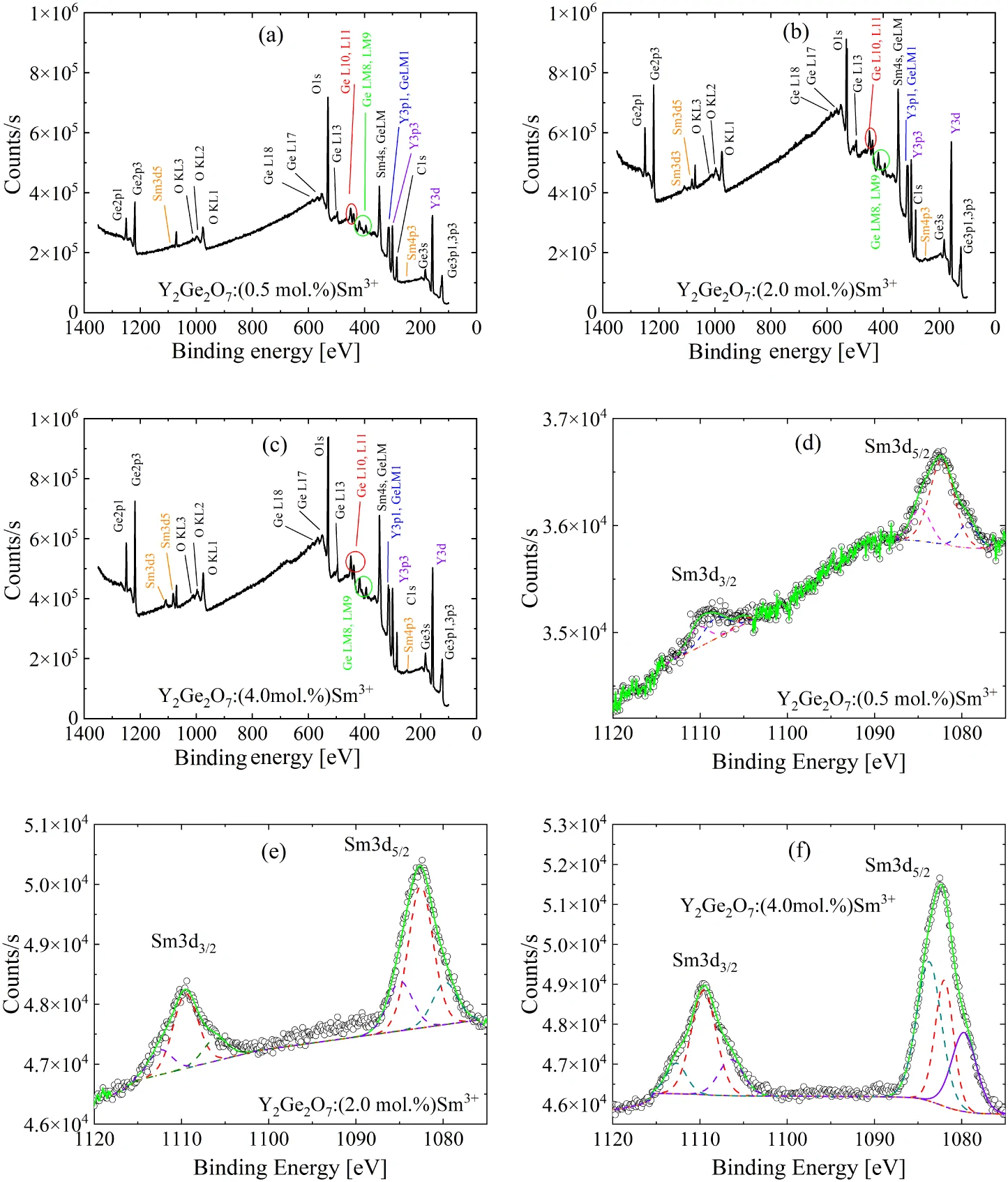
XPS spectra of Y_2_Ge_2_O_7_:Sm^3+^ phosphor: survey scan spectra of (a) 0.5 mol %, (b) 2.0 mol %, and (c) 4.0 mol %. The high-resolution XPS spectra for Sm 3d are shown in (d) 0.5 mol %, (e) 2.0 mol %, and (f) 4.0 mol %.

High-resolution XPS (HRXPS) spectra of Sm 3d_3/2_ and Sm 3d_5/2_ of these samples are presented in Figure 3d–f, where the bands are centered at ∼1109.7 eV and ∼1082.5 eV,^38^ respectively, being related to the Sm–O band.^10^ However, the spectral deconvolution yields three distinct components for each core level. Structurally, this is driven by the Sm^3+^ ions residing in highly distorted coordination polyhedra within Y_2_Ge_2_O_7_, or even because the pyrogermanate network has both highly polar terminal Ge–O^–^ bonds and bridging Ge–O–Ge bonds, the samarium ions interacting with them feel slightly different effective charges.^39,40^ Moreover, the primary Sm^3+^ peak can split via multiplet interactions into two tight subcomponents, while a third component appears at higher binding energy as a shakeup or charge-transfer satellite (often linked to Sm–O hybridization), thereby requiring multiple components to account for the asymmetric tailing and line broadening of the primary photoemission signal. Moreover, in Figure S2, we show the HRXPS spectra of Y, Ge, and O. The photoemission lines of Y^3+^ (Figure S2a,d,e) present in Y_2_Ge_2_O_7_ are centered at ∼159.9 eV and ∼156.9 eV, corresponding to Y 3d_3/2_ and Y 3d_5/2_, respectively. Regarding Ge 2p_3/2_ and Ge 2p_5/2_ (Figure S2b,e,h), the peaks are detected at ∼1218.9 eV and ∼1250.0 eV^34^. The O 1s_L_ and O 1s_S_ related to Y–O and Ge–O bonds and to −OH adsorbed on the surface appear at ∼529.8 eV and ∼530.7 eV, respectively, as shown in Figure S2c,f,i. These findings confirm the successful incorporation of Sm^3+^ into the Y_2_Ge_2_O_7_ host lattice.

### 3.2 Optical Spectroscopy

Figure 4a shows the diffuse reflectance spectra (DRS) of Y_2_Ge_2_O_7_:*x*Sm^3+^ phosphor recorded in the range of 175–1800 nm, using finely ground powder samples. All spectra exhibit a moderate diffuse reflectance signal ranging from 77% to 91%, and band-edge absorption at 200 nm. A number of sharp absorption lines corresponding to trivalent samarium transitions within the 4*f* ^5^ shell are observed in the spectra (inset of Figure 4a), starting from the ^6^ *H*_5/2_ ground state to ^4^ *G*_11/2_ (319.5 nm), ^4^ *H*_9/2_ (344.5 nm), ^4^ *D*_3/2_ (362 nm), ^4^ *P*_7/2_ (375.5 nm), ^4^ *F*_7/2_ (403.5 nm), ^4^ *I*_13/2_ (462 nm), ^4^ *I*_11/2_ (473 nm), ^6^ *F*_9/2_ (1078.5 nm), ^6^ *F*_7/2_ (1229 nm), ^6^ *F*_5/2_ (1370 nm), ^6^ *F*_3/2_ (1478.5 nm), and ^6^ *F*_1/2_ (1540.5 nm), respectively.^41–43^ From the DR spectra and using the Kubelka–Munk theory^44^ and the procedure described by Morales et al.,^45^ the optical band gap of Sm-doped Y_2_Ge_2_O_7_ phosphors was calculated. Figure 4b shows the Tauc graphs with (*F*(*R*_∞_) *hν*)^2^ versus photon energy (*hν*) for all samples; from these plots and extrapolating the linear fitted region to (*F*(*R*_∞_)*hν*)^2^ = 0, the band gap *E*_g_ was obtained. The values given in Table 1 were found to be nearly independent of the Sm^3+^ content, within experimental error. These results are similar to those recently reported for (Y_2–*x*_Tb_*x*_)­Ge_2_O_7_ samples, 6.13 ± 0.02 eV;^13^ (Y_2–*x*_Tm_*x*_)­Ge_2_O_7_ samples, 6.1 ± 0.1 eV,^38^ for Y_2–*x*_Ge_2_O_7_:0.005Pr^3+^, *E*_g_ = 6.03 eV,^11^ where *E*_g_ values also do not depend significantly on the impurity concentration, and those recently reported for Sr_2_Ga_2_GeO_7_ and Sr_2_Ga_2_GeO_7_:0.02Sm^3+^, 5.03 and 5.01 eV, respectively.^46^ On the other hand, these values are greater than those found for (Y_2‑x_Eu_*x*_)­Ge_2_O_7_ samples,^14^ which were prepared under the same method employed here, 4.0 eV (pure host) to 4.7 eV (4.0 mol %), for Tb_2_Ge_2_O_7_, 4.84 eV,^47^ and Y_2_Ge_2_O_7_:(1.0 mol %)­Nd^3+^, 3.99 eV ^12^.

**Figure 4:**
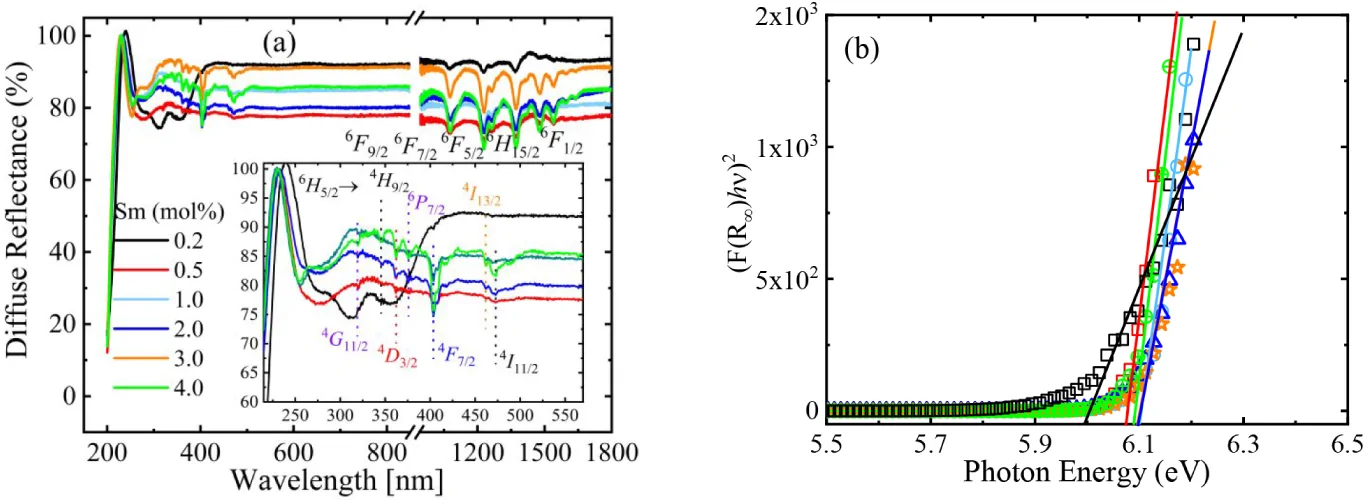
(a) Diffuse reflectance spectra; the inset shows the observed Sm^3+^ absorption bands, and (b) plots of (F­(R_∞_) × *h*ν)^2^ versus photon energy of Y_2_Ge_2_O_7_:*x*Sm^3+^ samples.

**Table 1:** Band Gap *E*_g_ Values for Y_2_Ge_2_O_7_:*x*RE^a^ ^3+^

*x* (mol %)	Sm^3+^	Tm^3+48^	Eu^3+14^	Tb^3+13^	Nd^3+12^	Pr^3+11^
0.2	5.99	6.1	4.2	6.13	-	
0.5	6.10	6.0	4.3	6.13	-	6.03
1.0	6.10	6.1	4.7	6.13	3.99	
2.0	6.10	6.0	4.6	6.13	3.99	
3.0	6.08	6.1	4.7	-	-	
4.0	6.07	6.0	4.7	6.13	-	

a (±0.05 eV).

### 3.3 Photoluminescence

Figure 5a shows the excitation spectra of Y_2_Ge_2_O_7_:Sm^3+^ doped with 0.2, 0.5, 1.0, 2.0, 3.0, and 4.0 mol % monitored at 601.6 nm (^5^ *G*_5/2_→^6^ *H*_7/2_) at room temperature. No evidence of a charge transfer band is observed in all spectra. A number of sharp bands between 325 and 550 nm were attributed to the intraconfigurational 4*f*-4*f* transitions of Sm^3+^ in the matrix, from the ^6^ *H*_5/2_ ground state to ^4^ *G*_9/2_ (332.5 nm), ^4^ *H*_9/2_ (344.5 nm), ^4^ *D*_3/2_ (361.3 nm), ^6^ *P*_7/2_ (373.9 nm), ^4^ *L*_15/2_ (390.7 nm), ^4^ *F*_7/2_ (402.7 nm), ^4^ *M*_19/2_ (407.5 nm), ^6^ *P*_5/2_ + ^4^ *P*_5/2_ (414 – 417.5 nm), ^6^ *P*_3/2_ (423.4 nm), ^4^ *G*_9/2_ (439.9 nm), ^4^ *I*_13/2_ (460.9 nm), ^4^ *I*_11/2_ (472 nm), ^4^ *I*_19/2_ (485.59 nm), ^4^ *G*_7/2_ (501.4 nm), ^4^ *F*_3/2_ (526.9 nm), and ^4^ *G*_5/2_ (546.7 nm),^41–43^ where the ^6^ *H*_5/2_→^4^ *F*_7/2_ transition is dominant independent of the samarium content, being, therefore, the chosen one for emission measurements of Y_2_Ge_2_O_7_:*x*Sm^3+^ phosphors.

**Figure 5:**
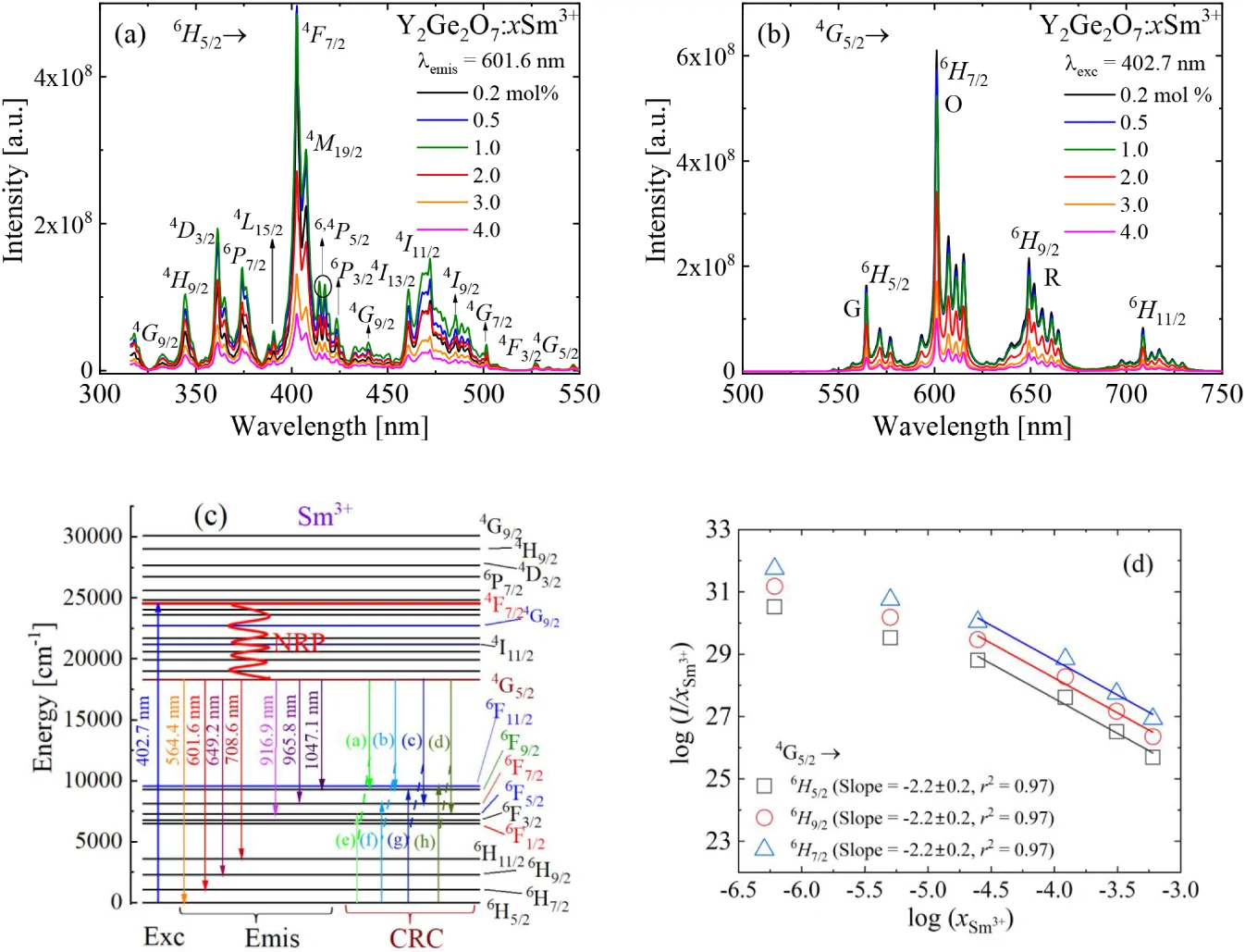
(a) Excitation spectra by monitoring the Sm^3+^ emission at 601.6 nm. (b) Emission spectra of Y_2_GeO_7_:*x*Sm^3+^ samples in the visible region. (c) Energy diagram of Sm^3+^ ion, the curved arrow represents the multiphonon relaxation, and the cross-relaxation process is assigned by straight arrows joined by dashed lines. (d) Curve of logI/*x* vs log*x* in Y_2_Ge_2_O_7_ phosphor (λ_exc_ = 402.7 nm). NRP is the nonradiative transition process.

Figure 5b shows the emission spectra of Y_2_Ge_2_O_7_:*x*Sm^3+^ phosphors under 402.7 nm wavelength excitation, presenting four groups of emission lines with main peaks at 564.6, 601.6, 649.2, and 708.6 nm, typical of Sm^3+^ transitions and assigned to transitions ^4^ *G*_5/2_ → ^6^ *H*_5/2_, ^4^ *G*_5/2_ → ^6^ *H*_7/2_, ^4^ *G*_5/2_ → ^6^ *H*_9/2_, and ^4^ *G*_5/2_ → ^6^ *H*_11/2_, respectively, that can be observed. All groups are split into a maximum number of (*J* + 1/2) components, indicating a high degree of crystallinity of the sample powders and that the Sm^3+^ ions are located in a low site symmetry in the Y_2_Ge_2_O_7_ matrix. The emission intensities decrease in the sequence of Sm3+ content: 0.2 > 0.5 > 1 > 2.0 > 3.0 > 4.0 mol %, indicating that the radiationless deactivation process is operative. Regardless of Sm^3+^ content, the dominant emission is a strong red emission due to the ^4^ *G*_5/2_ → ^6^ *H*_7/2_ transition. The emission bands have the same Stark splitting, position, and line width, indicating that Sm^3+^ occupies a similar site-symmetry environment in all studied samples. Figure S3 shows the integrated area of the ^4^ *G*_5/2_ → ^6^ *H*_7/2_ transition, where it is observed that it decreases monotonically with increasing Sm^3+^ content due to the concentration quenching effect. A similar result was observed for other trivalent samarium-doped oxide phosphors.^49–51^

In addition, to better understand the energy transfer process involved in the observed concentration quenching, the energy level diagram for Sm^3+^-doped Y_2_Ge_2_O_7_ is depicted in Figure 5c based on diffuse reflectance, PL excitation, and emission spectra. The level assignment of Sm^3+^ energy levels was based on refs. 41–43 . Upon 402.7 nm excitation, the samarium ions lying in the ^6^ *H*_5/2_ ground state are transferred to the ^4^ *F*_7/2_ excited state, where electronic vibrational relaxation occurs among the excited levels of Sm^3+^ ions, leading to a nonradiative process (NRP) giving rise to cross-relaxation (CR) processes displayed in Figure 5c, where the transitions labeled (a)–(d) are closely matched to the absorption transitions (e)–(h), respectively, and the radiative transitions occurring in the visible region from ^4^ *G*_5/2_ to ^6^ *H*_5/2,7/2,9/2,11/2_ levels.

Besides, according to Judd-Ofelt theory,^52,53^ the first one, ^4^ *G*_5/2_ → ^6^ *H*_5/2_ is a magnetic-dipole (MD) transition, while the third one, ^4^ *G*_5/2_ → ^6^ *H*_9/2_, is purely electric-dipole (ED), named green (**G**) and red (**R**) emissions, respectively, where the intensity ratio between them is useful to explain the symmetry of the local environment of the trivalent 4*f* ions, specifying the asymmetry nature of the host matrix, as well as the covalent/ionic bonding character. Moreover, the strong red-emitting transition ^4^ *G*_5/2_ → ^6^ *H*_7/2_ at 601.6 nm (Δ*J* = ± 1) is a partly magnetic dipole (MD) and partly electric dipole (ED) nature emission band (labeled orange, **O**). It can be observed that the **R**/**G** ratio (see Figure S3) was independent of Sm^3+^ concentration, ∼1.93, indicating the asymmetric nature of the host matrix and that the overall Sm^3+^ local environments do not change with different concentrations,^49^ in accordance with the crystal structure of Sm^3+^ in the Y^3+^ sites in Y_2_Ge_2_O_7_. Furthermore, the intensity of ^4^ *G*_5/2_ → ^6^ *H*_9/2_ (ED) transition is greater than that of the MD ^4^ *G*_5/2_ → ^6^ *H*_5/2_ transition, suggesting an asymmetric Y_2_Ge_2_O_7_ host matrix.^50^

In order to explore the observed concentration quenching mechanisms, the relationship between the Sm^3+^ content (*x* mol %) and the emission intensity (*I*) was discussed according to the Dexter model, using the formula:^54^

1 $$\log\left(\frac{I}{x_{Sm^{3+}}}\right)=A-\left(\frac{\theta }{3}\right)\log\left(x_{Sm^{3+}}\right)$$

where $x_{Sm^{3+}}$ is the concentration of the doping ion, *I* represents the integrated area intensity of the transition, θ is the multipolar parameter assuming 6, 8, and 10 for dipole–dipole, dipole–quadrupole, and quadrupole–quadrupole interactions, respectively, and *A* is a parameter of fit. The fitting results given in Figure 5d, with the slope value of about −2.23, give θ = 6.6 ± 0.6, indicating that the dipole–dipole interaction could be the major mechanism for concentration quenching of Sm^3+^-doped Y_2_Ge_2_O_7_ phosphor.

The effect of samarium content on the photometric parameters, such as CIE (Commission International d’Eclairage) (*x*,*y*) color coordinates, color purity (CP), and correlated color temperature (CCT), was also investigated. Concerning (*x*,*y*) color coordinates, the chromaticity diagram of CIE 1931 was calculated from emission spectra taken at room temperature and λ_exc_ = 402.7 nm, by using the methods defined by Wyman et al.,^55^ and presented in Figure 6a CIE, Figure 6 CCT and CP, and Table 2. The color purity (CP) was calculated by using eq 4, in order to quantify its chromaticity behavior:^56^

2 $$CP=\sqrt{\frac{{\left(x-x_{i}\right)}^{2}+{\left(y-y_{i}\right)}^{2}}{{\left(x_{d}-x_{i}\right)}^{2}+{\left(y_{d}-y_{i}\right)}^{2}}}\times 100\%$$

where (*x*,*y*) for each sample is given in Table 2, (*x*_i_,*y*_i_) = (0.333, 0.333) and (*x*_d_,*y*_d_) are, respectively, the color coordinates of the standard white light and the dominant wavelength point for red color (0.632, 0.368), the region where samarium ions typically emit.^56^ As can be seen in Table 2, the CIE (*x*,*y*) coordinates for Y_2_Ge_2_O_7_:*x*Sm^3+^ appear to be independent of Sm^3+^ concentration, lying in the red region (0.616, 0.382). The obtained CIE coordinates lie close to those of Y_2_O_3_:Eu^3+^ (0.62, 0.37),^57^ indicating that the studied samarium-doped yttrium germanate phosphor is a good candidate to combine with green and blue phosphors to generate white light, such as Y_2_Ge_2_O_7_:*x*Tb^3+13^. In addition, the Y_2_Ge_2_O_7_:*x*Sm^3+^ CP values are also nearly independent of samarium concentration, at 95.8% for all samples. The higher value of CP (∼96%) is comparable to those of Sm^3+^-doped SrTi­(PO_4_)_2_,^58^ La_10_W_22_O_81_ ^59^ and Y_2_Si_2_O_7_.^60^ This finding is also similar to that found for Y_2_Ge_2_O_7_:*x*Eu^3+14^ where CP values do not depend on Eu^3+^ content, differing from what is reported for Y_2_Ge_2_O_7_:*x*Tb^3+^, where the CP values increase from 19.2 to 94.1%, as the Tb^3+^ concentration grows from 0.5 to 4.0 mol %^13^ due, principally, to the presence of the emissions from the ^5^D_3_ energy level of terbium in the blue region, which decreases as CR becomes active at higher doping (see Figure 3b in ref^13^). Finally, the CCT values were calculated by using the McCamy formula:^61^ CCT = −437*n* ^3^ + 3601*n* ^2^ – 6861*n* + 5514.31, where *n* = (*x* – *x*_c_)/(*y* – *y*_c_), *x* and *y* being the chromaticity coordinates, and *x*_c_ = 0.3320 and *y*_c_ = 0.1858, the coordinates of the chromaticity epicenter. The values listed in Table 2 are nearly independent of the Sm^3+^ content, varying from 1807 to 1800 K for samples with 0.2 and 4.0 mol %, indicating that Y_2_Ge_2_O_7_:*x*Sm^3+^ has potential application in light devices.

**Figure 6:**
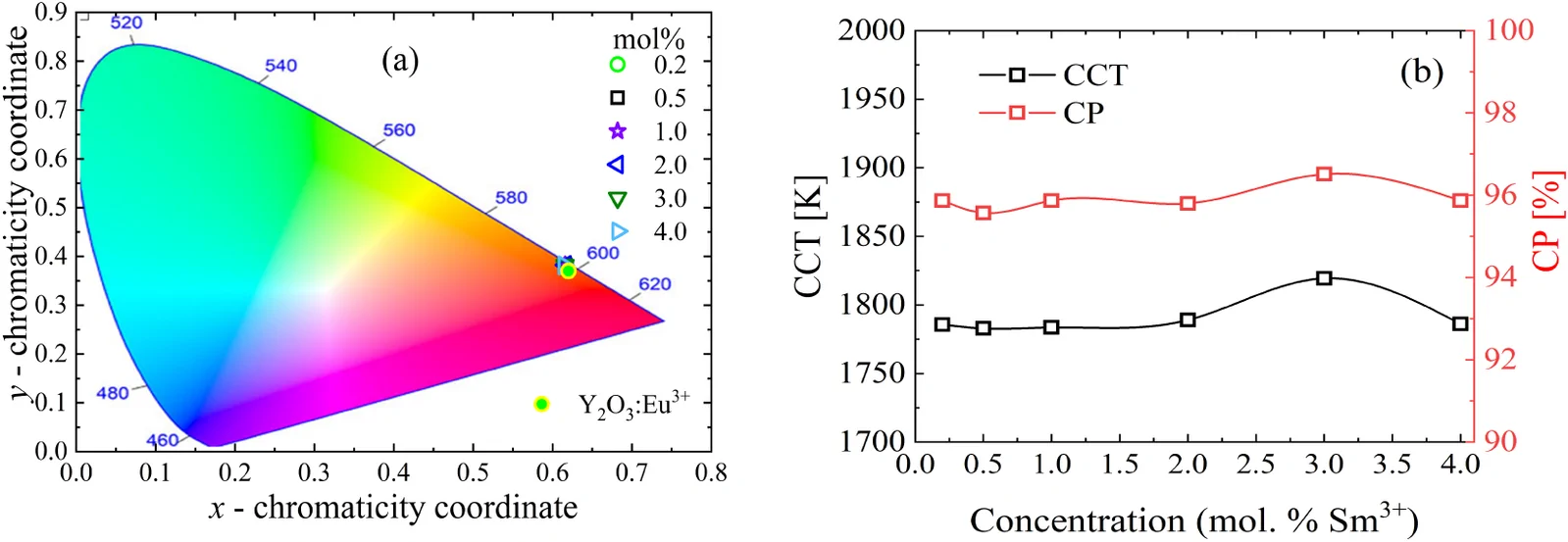
(a) CIE (*x*,*y*) coordinates for Y_2_Ge_2_O_7_:*x*Sm^3+^, and (b) CP and CCT values as a function of samarium concentration (λ_exc_ = 402.7 nm). In Figure 5a, the CIE for Y_2_O_3_:Eu^3+^ is (0.62, 0.37).

**Table 2:** Photometric Parameters: *R*/*G* and *O*/*G* Ratios, τ and a are, Respectively, the Experimental Lifetimes and the Amplitudes of the Fitted Decay Curves of the ^4^ *G*_5/2_ → ^6^ *H*_7/2_ Transition at 601 nm (λ_exc_ = 402.7 nm), CIE (*x,y*) Chromaticity Coordinates, Correlated Color Temperature (CCT), and Color Purity (CP)

Sm^3+^ (mol %)	*R*/*G*	*O*/*G*	*A*A_1_	τ_1_ (ms)	*A*A_2_	τ_2_ (ms)	τ_AV_ (ms)	(*x*,*y*)	CP (%)	CCT (K)
0.2	1.93	3.40	0.24	1.61(5)	0.76	2.23(2)	2.08(2)	(0.617, 0.382)	95.87	1785.68
0.5	1.93	3.40	0.14	1.08(2)	0.86	2.035(7)	1.90(2)	(0.616, 0.382)	95.57	1782.86
1.0	1.93	3.43	0.23	1.01(10)	0.77	1.996(6)	1.77(2)	(0.616, 0.382)	95.87	1783.58
2.0	1.93	3.42	0.33	0.636(5)	0.67	1.451(5)	1.18(2)	(0.616, 0.382)	95.8	1788.99
3.0	1.93	3.43	0.41	0.465(5)	0.59	1.145(7)	0.86(2)	(0.616, 0.382)	96.51	1819.42
4.0	1.93	3.44	0.43	0.305(5)	0.57	0.847(7)	0.61(2)	(0.614, 0.381)	95.87	1786.09

### 3.4 Electronic Structure Calculations

The electronic structures of pristine and Sm-doped Y_2_Ge_2_O_7_ were investigated to elucidate the impact of rare-earth incorporation on the host electronic states and their relation to the optical properties discussed above. The total and projected density of states (DOS/PDOS), calculated within the DFT-1/2 framework to correct the bandgap underestimation of semilocal functionals, are shown in Figure 7.

**Figure 7:**
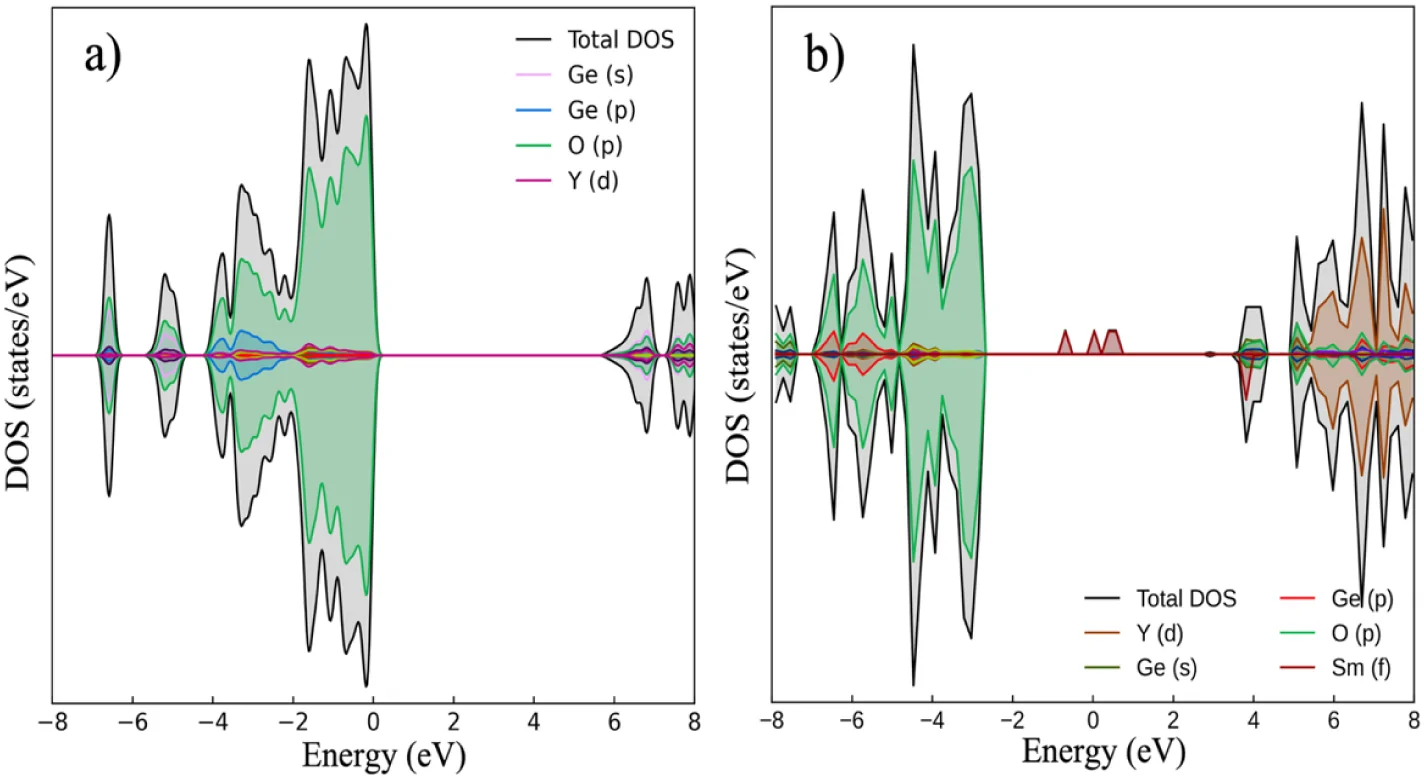
Calculated total and atom- and orbital-resolved density of states (DOS) for (a) pristine Y_2_Ge_2_O_7_ and (b) Sm-doped Y_2_Ge_2_O_7_ calculated within the DFT-1/2 method. The Fermi level is set at zero energy. The shaded gray areas represent the total DOS, while colored lines correspond to the projections onto the Ge (s and p), O (p), Y (d), and Sm (f) orbitals.

For the pristine compound (Figure 7a), the valence band (VB), extending from approximately −8 eV up to the valence band maximum, is predominantly composed of O 2p states, with minor contributions from Ge 4p and Y 4d orbitals. This hybridization indicates a partial covalent character of the M–O (M = Y, Ge) bonds, consistent with the structural features of the host matrix. The conduction band (CB) is mainly formed by unoccupied Y 4d and Ge 4p states. The fundamental direct bandgap (see Figure S4), extracted from the calculated band structure, is found to be 5.75 eV. This result is in reasonable agreement with the optical bandgap values (∼5.99–6.10 eV) obtained from the diffuse reflectance spectra (Figure 4), confirming the reliability of the DFT-1/2 approach.

Upon Sm incorporation (Figure 7b), the overall DOS profile remains essentially unchanged, indicating that the host electronic structure is preserved upon substitution. However, the introduction of Sm gives rise to highly localized 4f states within the band gap. These states appear as sharp features, with occupied levels located near the valence band maximum and unoccupied levels positioned below the conduction band minimum, leading to the formation of intragap states. Furthermore, a comparison of the DOS for intermediate (1 Sm, *x* = 1.5625 mol %) and high (2 Sm, *x* = 3.125 mol %) dopant concentrations is provided in Figure S5, confirming that this localized electronic picture and the fundamental host bandgap remain invariant regardless of the doping level. Additionally, the energy degeneracy between ferromagnetic and antiferromagnetic configurations in the 2 Sm model demonstrates a negligible interdopant interaction, confirming that the Sm^3+^ ions act as isolated centers.

The presence of these localized Sm 4f states is consistent with the optical response of the material. The large bandgap of the Y_2_Ge_2_O_7_ host (∼6 eV), determined from diffuse reflectance measurements (Figure 4), indicates that the matrix is transparent in the visible region. Accordingly, the photoluminescence spectra (Figure 4) exhibit only sharp emission lines associated with intra4f transitions of Sm^3+^ ions, without evidence of host-related emission. This behavior reflects the highly localized nature of the 4f electronic states, which are weakly coupled to the lattice and give rise to the characteristic narrow luminescence bands observed experimentally.

### 3.5 Decay Profiles, Energy Transfer Mechanisms, and Quantum Yield

The fluorescence decay curves of the Sm^3+^ ions in the host Y_2_Ge_2_O_7_:*x*Sm^3+^ phosphors were measured by monitoring the ^4^ *G*_5/2_ → ^6^ *H*_7/2_ emission at 601.0 nm with λ_exc_ = 402.7 nm; the results for samples with doping levels of 0.2 to 4.0 mol %, are presented in Figure 8a. In all cases, the curves were best fitted using the biexponential expression:^62^

3 $$I\left(t\right)=y_{0}+A_{1}\exp\left(-t/\tau_{1}\right)+A_{2}\exp\left(-t/\tau_{2}\right)$$

where τ_1_ and τ_2_ are decay times, which determine the decay rate for corresponding exponential components, *A*_1_ and *A*_2_ are the amplitudes or contributions of each lifetime, and *y*_0_ is the intensity at time *t* = 0. The average lifetimes were calculated by using the equation:^62^

4 $$\tau_{AV}=\left(A_{1}{\tau_{1}}^{2}+A_{2}{\tau_{2}}^{2}\right)/\left(A_{1}\tau_{1}+A_{2}\tau_{2}\right)$$

**Figure 8:**
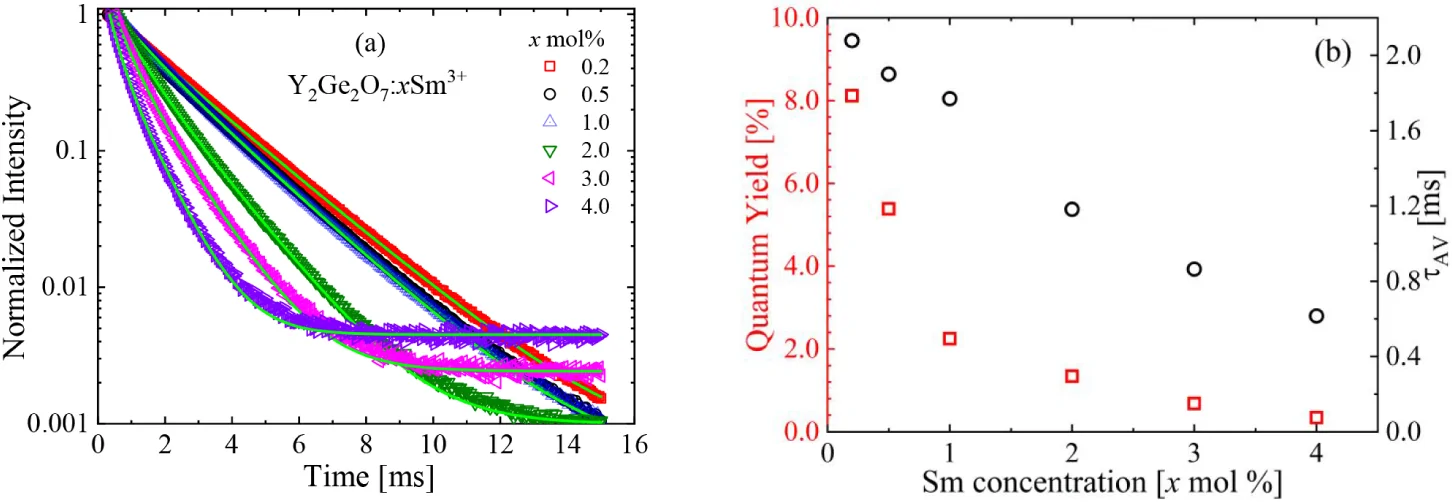
(a) Normalized emission decay curves of Y_2_Ge_2_O_7_:*x*Sm^3+^ samples (*x* = 0.2, 0.5, 1.0, 2.0, 3.0, and 4.0 mol %), by monitoring the ^4^ *G*_5/2_ → ^6^ *H*_7/2_ transition at 601.6 nm with excitation at 402.7 nm. Symbols and lines represent the experimental data and the fit with eq 2, respectively. *r*_adj_^2^ = 0.999. (b) iQY and average lifetime (τ_AV_) evolutions as a function of Sm^3+^ concentration.

The τ and *A* values at different temperatures are given in Table 2.

It can be observed in the decay values that, within the experimental error, the average decay time (τ_AV_) decreases monotonically from 2.08 to 0.61 ms, as Sm^3+^ concentration increases from 0.2 up to 4.0 mol % (Figure 8b and Table 2), which can be attributed to the occurrence of a quenching process between Sm^3+^ ions in the Y_2_Ge_2_O_7_ host, due to the decreased distance between Sm^3+^ ions, which enhances the energy transfer between the Sm^3+^ ions through the nonradiative transition, as observed in Sm^3+^-doped Mg_3_Gd_2_Ge_3_O_12_.^63^ The biexponential decay results indicate that, in these compounds, two decay mechanisms are involved in the observed emissions, as reported by Priya et al.^64^ Similar findings were found in the case of Y_2_Ge_2_O_7_:*x*Eu^3+14^, where the decay lifetime was also found to be biexponential, being correlated to europium ions located in microenvironments with different symmetries, presenting more than one channel of deactivation. Our findings are also in good agreement with those reported in the literature for other trivalent samarium-doped germanate phosphors, such as Li_2_MGeO_4_ (M = Zn, Mg) doped with 0.5 mol % of Sm^3+^, which exhibits 1.36 and 1.55 ms, respectively,^65^ and with those found for YInGe_2_O_7_:Sm^3+^ where the lifetime decreases from 4.5 to 0.8 ms as Sm^3+^ ion content grows from 1 to 20 mol %.^66^

The dependence of decay values with the Sm^3+^ concentration can be explained by the Van Uitert model:^67^

5 $$\tau \left(x\right)=\tau_{0}/\left\{1+{\left(x/x_{0}\right)}^{\theta /3}\right\}$$

where τ­(*x*) is the average fluorescence lifetime at concentration *x*, τ_0_ is the lifetime of the emission level under dilute condition, *x*_0_ is the critical concentration at which the lifetime of the sample is half of the intrinsic one, θ has the same physical meaning in eq 1. The fitting results given in Figure 9a support the conclusion that Sm^3+^ is involved in dipole–dipole interaction, in accordance with what was found above (Figure 5d) for intensity concentration quenching, where the best parameters are θ = 5.4 ± 0.6, *x*_0_ = 2.4 ± 0.1 mol %, and τ_0_ = 2.1 ± 0.5 ms. On the other hand, based on the lifetime results, the cross-relaxation rate (*W*_Sm–Sm_) and efficiency (η_Sm–Sm_) of this process are quantified using the equations:^68^

6 $$W_{Sm-Sm}=\frac{1}{\tau_{Sm-Sm}}-\frac{1}{\tau_{0}}$$

7 $$\eta_{Sm-Sm}=1-\frac{\tau_{Sm-Sm}}{\tau_{0}}$$

where τ_0_ is the lifetime of the excited state under diluted conditions obtained from Van Uitert’s adjustment, and τ_Sm–Sm_ is given by the experimental data. As shown in Figure 9b, the cross-relaxation rates increase rapidly in the 0.2–4 mol % interval, achieving an efficiency of approximately 73% at 4 mol %. The cross-relaxation rate for the higher-doped Y_2_Ge_2_O_7_:Sm^3+^ sample is 1.2·10^3^ s^–1^, being the same magnitude as that estimated for YAM:Sm3+^69^ and YAP:Sm^70^ ^3+^, but lower than that reported in Li_(x)_Na_(1‑x)_Sm­(PO_3_)_4_.^71^ Moreover, a fit of *W*_Sm–Sm_ plot returns a slope close to two (blue line in Figure 9b), an indication that the weak quenching approximation is still valid below this concentration.^70^

**Figure 9:**
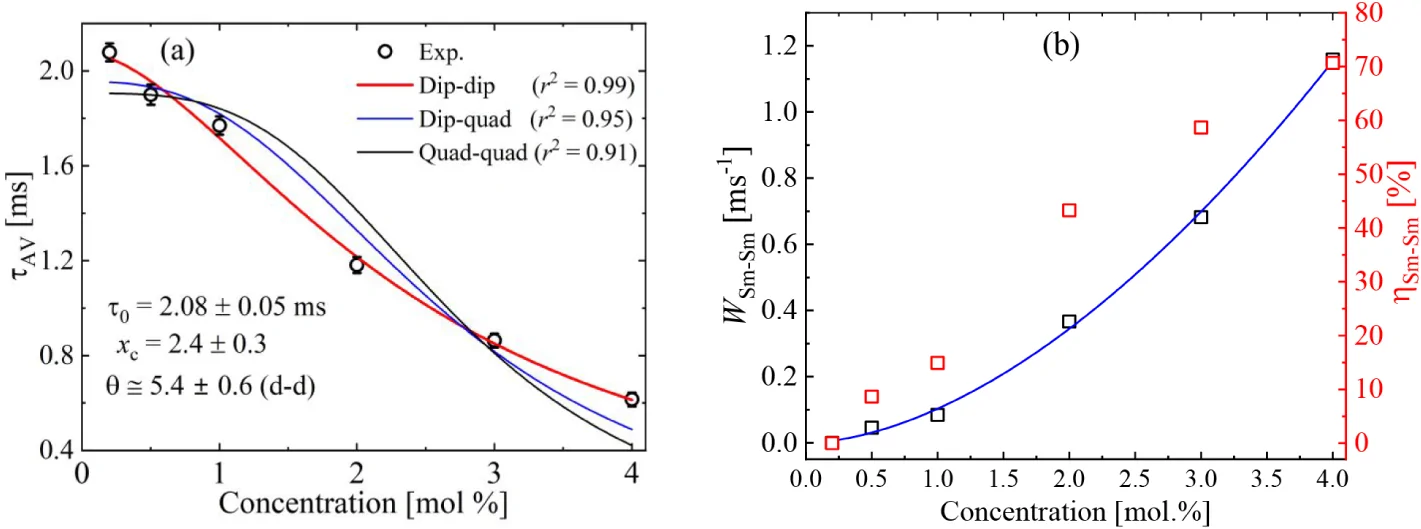
(a) Dependence of lifetime of ^4^ *G*_5/2_ energy level on doping Sm^3+^ concentration, solid lines are the fitting curves based on the Van Uitert’s model given in eq 5. (b) Quenching rates *W*_Sm–Sm_ (black) and efficiency (η_Sm–Sm_) for the ^4^G_5/2_ state calculated according to eqs 6 and 7, respectively, are plotted as a function of concentration. The blue line in (b) represents a fit with the *y* = *a*.*x* ^b^ function, with *b* = 1.74.

In order to investigate the PL efficiency of the Y_2_Ge_2_O_7_ powders, we measured the internal and external quantum yields (iQY and eQY).^32^ The iQY values are presented in Table 3 and in Figure 8b, while the eQY values are given in Table S2. It was observed that the *iQY* values decrease with increasing Sm^3+^ content, from 8.1% to 0.3% for samples with 0.2 and 4.0 mol %, respectively, following the same trend as the decay lifetimes (Figure 8b). The same behavior is followed by the eQY values (see Table S2). These results are in complete agreement with the analysis of concentration quenching by the Dexter model, as described above. Moreover, the decrease in lifetime values and the lowering of quantum yields (emission quenching) with an increasing Sm^3+^ content can be attributed to energy transfer via cross-relaxation during the stimulated emission process.

**Table 3:** Photoluminescent Internal Quantum Yields iQY (%) for Rare-Earth-Doped Y_2_Ge_2_O_7_ Matrix and for SFB:*x*Sm^3+^ Glass

RE^3+^(mol %)	Sm^3+^	Tm^3+48^	Tb^3+13^	Eu^3+ 14^	SFB:*x*Sm^3+^ (mol %)^43^	η_QY_ (%)^43^
0.2	8.1	8.0	0.5	1.2	0.01	1.04
0.5	5.4	10.8	6.6	0.1	0.1	0.99
1.0	2.2	5.3	4.8	2.7	0.5	0.74
2.0	1.3	2.7	9.4	3.6	1.0	0.60
3.0	0.7	1.3	–	7.2	2.0	0.48
4.0	0.3	0.7	10.0	4.5	4.0	0.22

Moreover, the large energy gap between the emitter level ^4^ *G*_5/2_ and the first energy level below, ^6^ *F*_11/2_, *ca* 8742 cm^–1^ (see Figure 5c), which is 10.3 times the matrix phonon energy, ∼845 cm^–1 14^, hinders the occurrence of multiphonon nonradiative decay.^43^ Therefore, the cross-relaxation mechanism is the main pathway for nonradiative rates. On the other hand, the observed behavior of *iQY* vs *x* shown in Figure 8b and Table 3 is similar to that reported for Tm^3+^,^48^ but opposite to that observed for Eu^3+14^ and Tb^3+13^ ions doped in Y_2_Ge_2_O_7_ with the same molar concentrations used here, i.e., in those cases iQY increases with the increase of the terbium or europium content. This is explained by the higher occurrence of the CR channels in the emitter level in Tm^3+^ (^1^ *D*_2_) and Sm^3+^ (^4^ *G*_5/2_), whereas the emitter level in the Tb^3+^ (^5^ *D*_4_) and Eu^3+^ (^5^ *D*_0_) ions does not present a cross-relaxation process, but follows the same trend for the fluorescence quantum yield (η_QY_, see ref.^43^ for definition) measured for Sm^3+^ ions in sodium fluoroborate (SFB) glasses.

### 3.6 Temperature-Dependent Luminescence

The study of the dependence of some photoluminescence parameters, such as the integrated intensity of the area of the emission bands, is one of the most important steps to evaluate the possibility of using the phosphor in optical devices or as thermosensors.^72–75^ So, in this work, the emission behavior as a function of temperature was investigated in the range of 293 to 332.6 K for all synthesized samples, and the spectra are shown in Figures 10a and S6. All of them present similar profiles of Sm^3+^ emission bands, consistent with those presented in Figure 5b, including the Stark splitting. The observed intensity decrease with increasing temperature is attributed to the thermal quenching effect. All samples present good thermal stability; for example, in the sample with 0.2 mol %, the PL intensity of emission at 601.6 nm (^4^ *G*_5/2_ → ^6^ *H*_7/2_) measured at 293 K compared to that at 332.6 K was only reduced by 11.1%. So, the PL intensity shows no obvious change with increasing temperature, as shown in Figures 10a and S6. However, it could be noted that the behavior of the intensity ratios **R/G** and **O/G** (Figure S7) with temperature was also analyzed, and they are nearly temperature-independent, showing that both the bonding character and Sm^3+^ site symmetry are unaffected in the temperature range analyzed.

**Figure 10:**
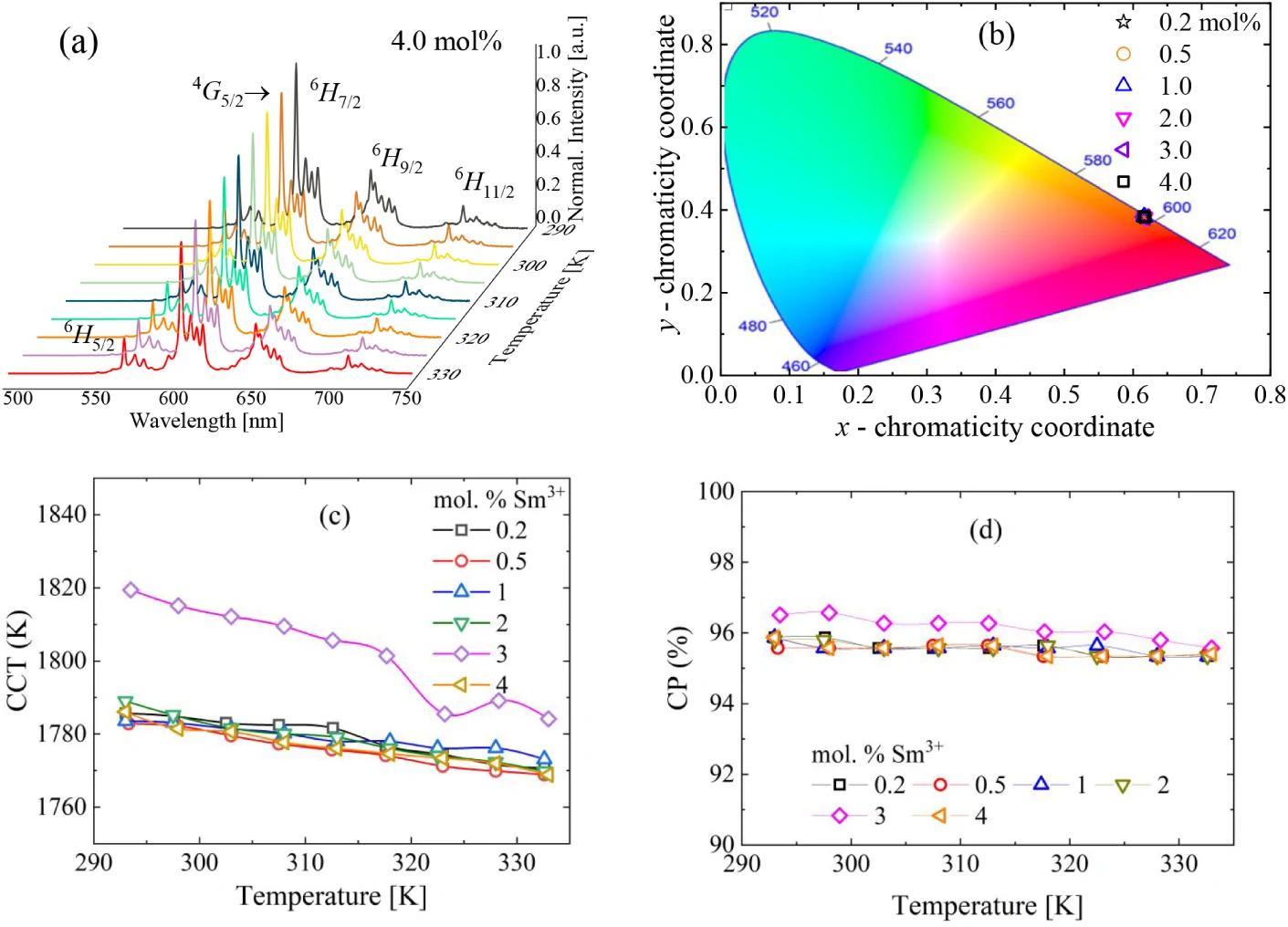
(a) Temperature dependence of the emission spectra of Y_2_Ge_2_O_7_:(4.0 mol %)­Sm^3+^, λ_exc_ = 402.7 mn. Thermal dependence of (b) CIE 1934 (*x,y*) color coordinates, (c) CCT, and (d) CP values of Y_2_Ge_2_O_7_:*x*Sm^3+^ phosphor.

From the spectra depicted in Figures 10a and S6, the thermal dependence of CIE (*x*,*y*) coordinates, CCT, and CP values was calculated, and the results are given in Figure 10b–10d, respectively. It was noted that the CIE color coordinates do not shift with temperature, remaining in the reddish region. On the other hand, the values of the CCT of all samples exhibit a small decrease (*ca*. 1%) when the temperature of the sample was increased, as shown in Figure 10c, indicating that CCT values have a small influence from the temperature in the Y_2_Ge_2_O_7_ matrix, as well as the ratio between the integrated area of the green component and those of the red and orange components, **R/G** and **O/G**, illustrated in Figure S7, remains almost constant as the temperature rises, independent of Sm^3+^ content. In addition, the CP values also do not change with increasing temperature (see Figure 10d) and are estimated to be close to 96% for all samples, larger than those reported for Sr_2_Ga_2_GeO_7_:0.02Sm^3+^ (80.8%),^46^ and for Y_2_MgTiO_6_:Sm^3+^ (81.9–84.0%).^10^ The obtained high CP and low CCT values are an indicative that the Y_2_Ge_2_O_7_:Sm^3+^ phosphor is a promising candidate for red-light-emitting phosphors.

For the optical thermometry approach, the intensity ratio of two energy transitions from the same excited energy level (^4^ *G*_5/2_) of Sm^3+^ as a function of temperature was used to verify the potential use of the Y_2_Ge_2_O_7_:*x*Sm^3+^ as ratiometric noncontact thermosensors based on sub-Stark level emissions instead of the integrated total area of emission. Figure 11a,b shows the temperature effect on the luminescence intensity ratios (Δ_1_ = *I*_3_/*I*_1_) (see Figure S8) between integrated areas of sub-Stark levels of emissions centered at 649.3 nm (^4^ *G*_5/2_ → ^6^ *H*_9/2_) and 564.7 nm (^4^ *G*_5/2_ → ^6^ *H*_5/2_) for Y_2_Ge_2_O_7_:(0.2 mol %)­Sm^3+^ and Y_2_Ge_2_O_7_:(4.0 mol %)­Sm^3+^, respectively. It was observed that Δ_1_ increases linearly with temperature and is fitted to the equation Δ_1_(*T*) = *aT* + *b*, where *a* is related to the absolute sensitivity, *b* is a constant, and *T* represents the absolute temperature. The relative sensitivities (*S*_R_) were calculated using the equation: *S*_*R*_ = |(∂Δ/∂*T*)/Δ| × 100%, where ∂R is the variation of the thermometric parameter for a certain temperature variation (∂*T*). On the other hand, the temperature resolution δ*T* is given by δ*T* = (1/*S*_R_)­(δΔ/Δ), where δΔ/Δ = 0.03% is the relative uncertainty for the photomultiplier tube (PMT).^73^ Within the several ratios Δ_1_ analyzed between the intensities of all emissions observed in the spectra, by following the procedure described in ref.^76^. The best-fitting results are presented in Figure 11a and 11b, together with the dependence of *S*_R_ on T shown in Figure 11c. The maximum *S*_R_ values are 0.21 and 0.23% K^–1^ at 293 K (20 °C), respectively, for the samples with 0.2 and 4.0 mol %, with their respective mean δ*T* values being 0.14 and 0.13 K for samples with 0.2 and 4.0 mol % also at 293 K, are listed in Table 4, where they are compared with the values for other Sm^3+^- doped oxide phosphors, with good potential for temperature sensing applications.^9^

**Figure 11:**
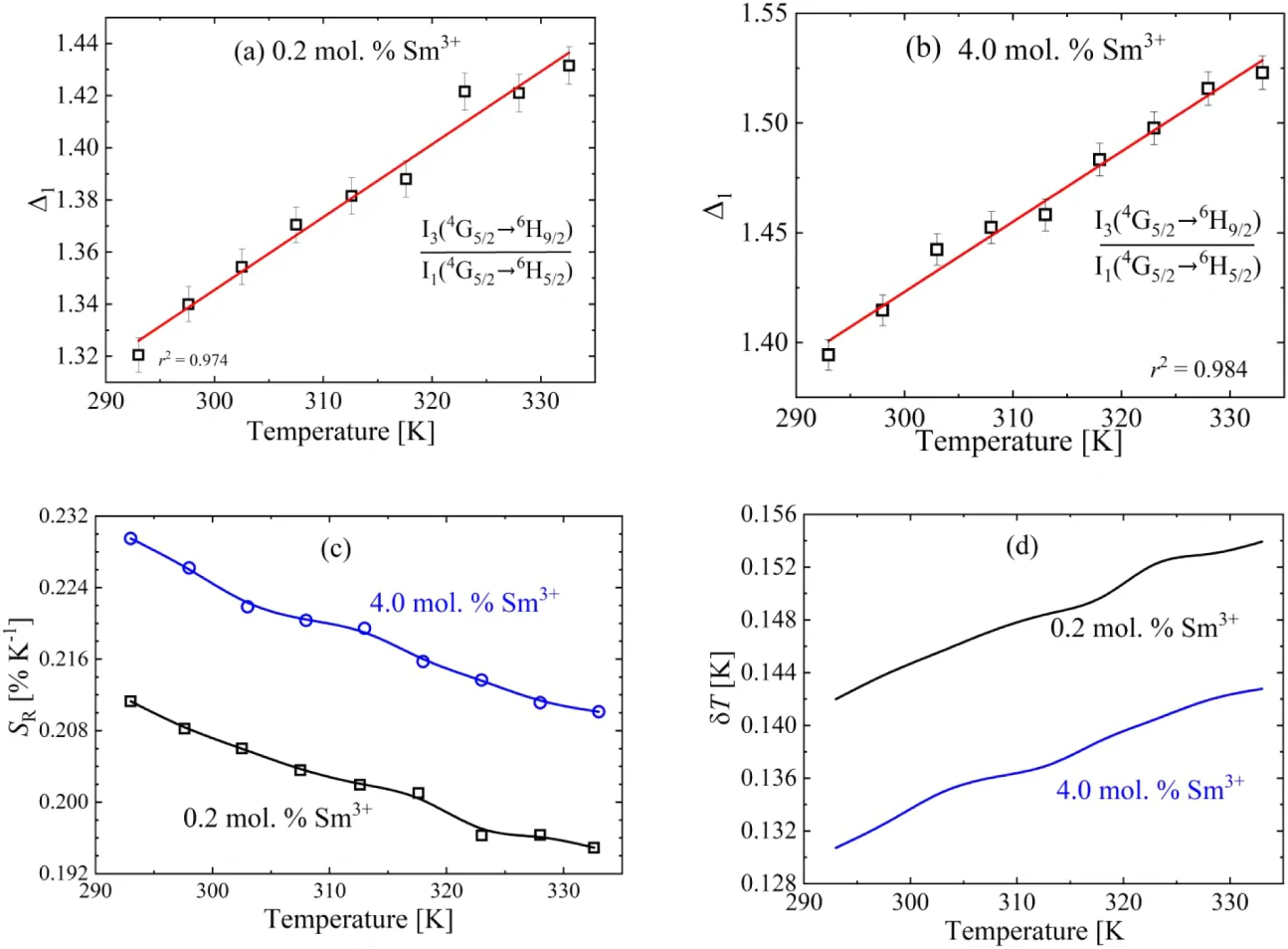
Thermal dependence of the ratio between integrated areas of Sm^3+^ sub-Stark levels of emissions centered at 649.3 nm (^4^ *G*_5/2_ → ^6^ *H*_9/2_) and 564.7 nm (^4^ *G*_5/2_ → ^6^ *H*_5/2_), for samples doped with (a) 0.2 and (b) 4.0 mol % Sm^3+^. The black symbols are the experimental data, and the red lines represent the fit as described in the text. Temperature dependence of (c) Sr and (d) δ*T* values for both samples. (d) In (c) the lines are a guide to the eyes.

**Table 4:** Sm^3+^-Doped Inorganic Compounds for Optical Thermometry Using the Red-To-Green Ratio as a Ratiometric Parameter^a^

Optical material	Temperature range (K)	Maximal *S*_R_ value (% K^–1^)	refs
Y_2_Ge_2_O_7_	293–339 K	0.230 @ 293 K	t.w.
SrMoO_4_	273–573 K	0.209 @ 458 K	^8^
Y_2_MgTiO_6_	298–498 K	0.260 @ 298 K	^10^
Ca_2_LaNbO_6_	313–573 K	0.220 @ 313 K	^9^

a n.a. not available.

Compared with other Sm^3+^-doped hosts that used the same **R/G** luminescent ratio for thermometric calculations, notably those with relative sensitivities exceeding 0.3% K^–1^, even when the temperature range exceeds 500 K. Therefore, our *S*_R_ results fall within the expected range for this metric in Sm^3+^-doped nano- and microcrystal oxides. On the other hand, it should be noted that cross-relaxation increases the *S*_R_ obtained in this temperature interval, even though this Δ_1_ ratio is not directly related to CR channels since both emissions originate from the same level (^4^ *G*_5/2_). Likely, the CR process increases the nonradiative rates in the ^4^ *G*_5/2_ level at higher-doped samples, which makes the samples more sensitive to temperature changes at mild temperatures. Therefore, these findings indicate that in our case, the CR process can act as an additional nonradiative channel for improving temperature sensing performance. Expanding to comparisons with other lanthanide-doped oxides, the higher *S*_R_ are found when the emissions used for the luminescence ratio belong to energy levels involved in the CR process, where one level is degraded by the CR process, whereas another level is favored by this process.^63,77^

## 4 Conclusions

In this paper, Y_2_Ge_2_O_7_:*x*Sm^3+^ (*x* = 0.2, 0.5, 1.0, 2.0, 3.0, and 4.0 mol %) phosphors were successfully synthesized by a sol–gel method. The formation of a single-phase structure was confirmed by PXRD. Subsequent morphological analyses via SEM and TEM revealed that the synthesized samples consist of agglomerated and polydisperse nanoscale particles. In addition, the elemental composition and chemical valence states of the synthesized Y_2_Ge_2_O_7_:Sm^3+^ phosphor were validated via XPS measurements. Regardless of the dopant concentration, all observed emission bands in the UV–vis region originate from the Sm^3+ 4^ *G*_5/2_ → ^6^ *H*_5/2_,_7/2,9/2,11/2_ electronic transitions, exhibiting well-resolved Stark splitting due to the low local site symmetry of the Sm^3+^ ions within the Y_2_Ge_2_O_7_ host. An optimal Sm^3+^ dopant concentration of ∼0.2 mol % was identified for the Y_2_Ge_2_O_7_:*x*Sm^3+^ matrix. The resulting high emission intensity ratios (**R/G** = 1.92; **O/G** = 3.45) underpin the pronounced red/orange luminescence of the phosphor. The energy transfer process was studied, and the cross-relaxation rate (*W*_Sm–Sm_) and efficiency (η_Sm–Sm_) were determined, which are of the order of 1.2·10^3^ s^–1^ and 73%, respectively, for a sample with 4.0 mol % of Sm^3+^. Moreover, the study of lifetime as a function of Sm^3+^ concentration revealed a dipole–dipole interaction among these ions. The CIE coordinates are nearly independent of both concentration and temperature, standing very close to those of the commercially available red phosphor Y_2_O_3_:Eu^3+^, indicating that this material can generate strong and vivid orange light even at low concentrations when excited at 402.7 nm. Furthermore, a decrease of QY with Sm^3+^ concentration, with a maximum value of 8.1% for the sample with 0.2 mol %, close to the best values found for this germanate phosphor doped with Tb^3+^ or Eu^3+^ ions was observed. In addition, temperature sensing performances were characterized by luminescence intensity ratios based on nonthermal coupled energy levels of Sm^3+^ ions, reaching a maximum relative sensing of 0.23%K^–1^ at 298 K, demonstrating its potential use as an optical thermometer.

## Supplementary Material



## Data Availability

The data that support the findings of this study are available from the corresponding author upon reasonable request.

## Notes

Supplementary figures of structural (EDS and SEM images, XPS spectra), optical, theoretical (electronic band structure and density of states), temperature emission spectra, and tables (reagents amounts, eQY, CIE, and CCT values) (PDF)
