# Supplementary material for: Sm3+-Doped Y2Ge2O7 Orange-Red Phosphor Prepared via the Sol–Gel Method for Multimodal Applications: A Study of of the Effect of Dopant Concentration on Structural and Optical Properties
Source: ACS Omega. 2026 Oct 13;11(42):64870–83. doi: 10.1021/acsomega.6c05178 (PMC13717785; doi:10.1021/acsomega.6c05178)
Supplement: Supplementary file 1 [file ao6c05178_si_001.pdf]

Supporting Information

Sm^{3+} doped $Y_2Ge_2O_7$ orange-red phosphor prepared via sol-gel method for multimodal applications. A study of concentration doping on structural and optical properties

Amanda Alencar Arrais de Garcia [1], Isabella Lima Caetano[1], Francisco Alisson da Silva Torquato[1], Marcelo Marques[2], Lara K. Teles[2], Renato Borges Pontes[1], Lauro June Queiroz Maia[1], Ricardo Costa de Santana[1,*]

[1] *Grupo Física de Materiais (GFM), Instituto de Física, Universidade Federal de Goiás, Avenida Esperança s/n, Campus Samambaia, Goiânia - GO, CEP 74001-970, Brazil*
[2]*Group of Semiconductor Materials and Nanotechnology (GMSN), Aeronautics Institute of Technology (ITA), 12228-900 São José dos Campos (SP), Brazil*

**Corresponding author e-mail: santana@ufg.br*

Contents

1 Synthesis
Table S1……………………… 2

2 Structural characterizations
Figure S1 ……………………. 2
Figure S2 ……………………. 3

3 Optical characterization and Theoretical calculations
Figure S3 ……………………. 5
Figure S4 ……………………. 5
Figure S5 ……………………. 6
Table S2 …………………….. 7

5 Thermometric characterizations
Figure S6 ……………………. 7
Figure S7 ……………………. 8
Figure S8 ……………………. 9
Table S3 …………………….. 9
Table S4 ……………………. 10

References 10

Table S1. Reagents amounts in grams (g) used in the synthesis.

***x* % of Sm**	**GeO_2**	**$Y(NO_3)_3.6H_2O$**	**$Sm(NO_3)_3.6H_20$**	**$C_6H_8O_7.H_2O$**
0.2	0.2408	0.8779	0.00414	2.6469
0.5	0.2400	0.8705	0.0132	2.6425
1.0	0.2381	0.8591	0.0206	2.6603
2.0	0.2387	0.8366	0.0409	2.6203
3.0	0.2350	0.8125	0.0619	2.6082
4.0	0.2360	0.7985	0.0804	2.5921

Figure S1. SEM images and EDS mapping for $Y_2Ge_2O_7$:$x$$Sm^{3+}$ powders (a) 0.2 mol%, (b) 0.5 mol%, (c) 1.0 mol%, (d) 2.0 mol%, (e) 3.0 mol%, (f) 4.0 mol%, respectively. Amplification x5000, 5nm of resolution.

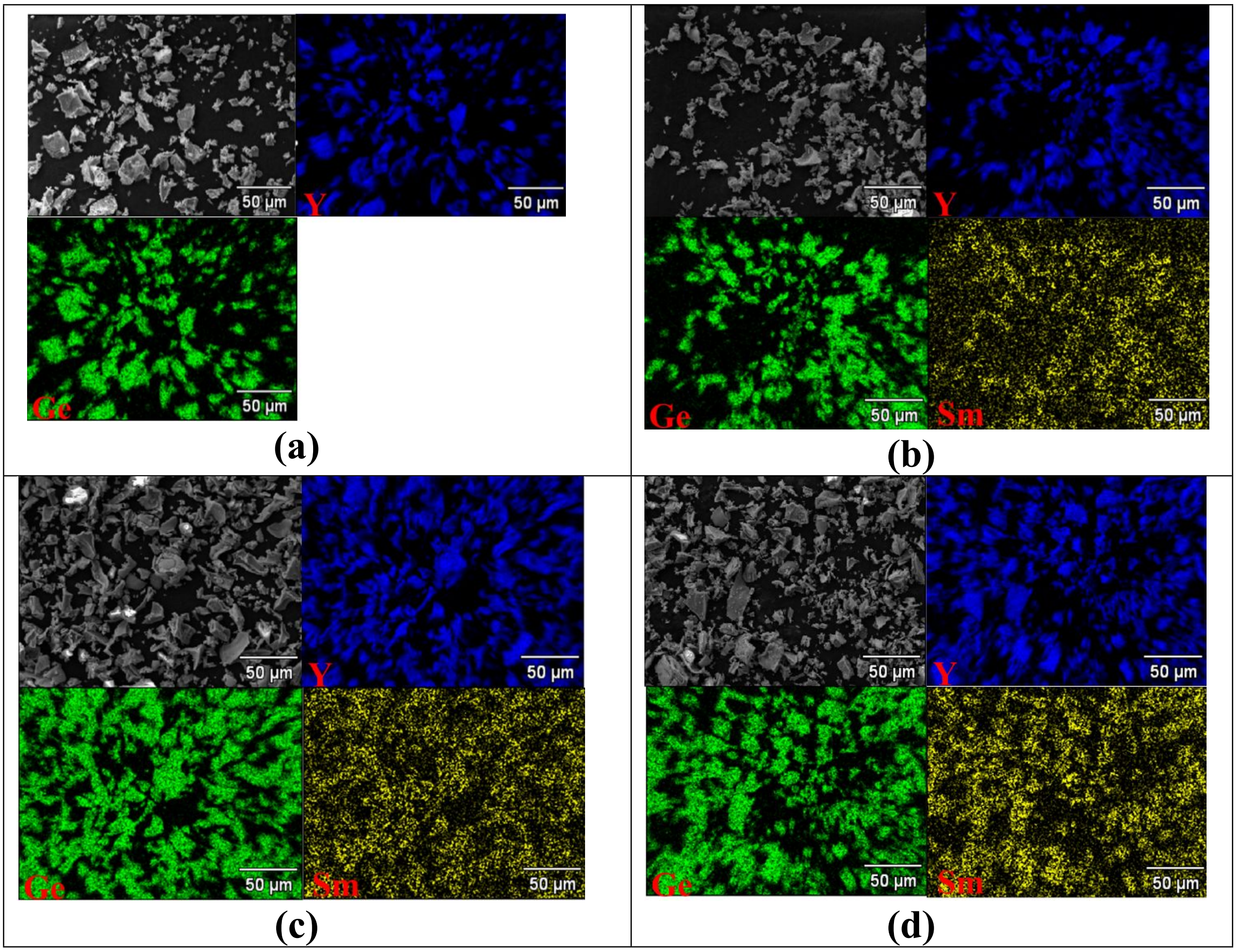

(a) (b) (c) (d)

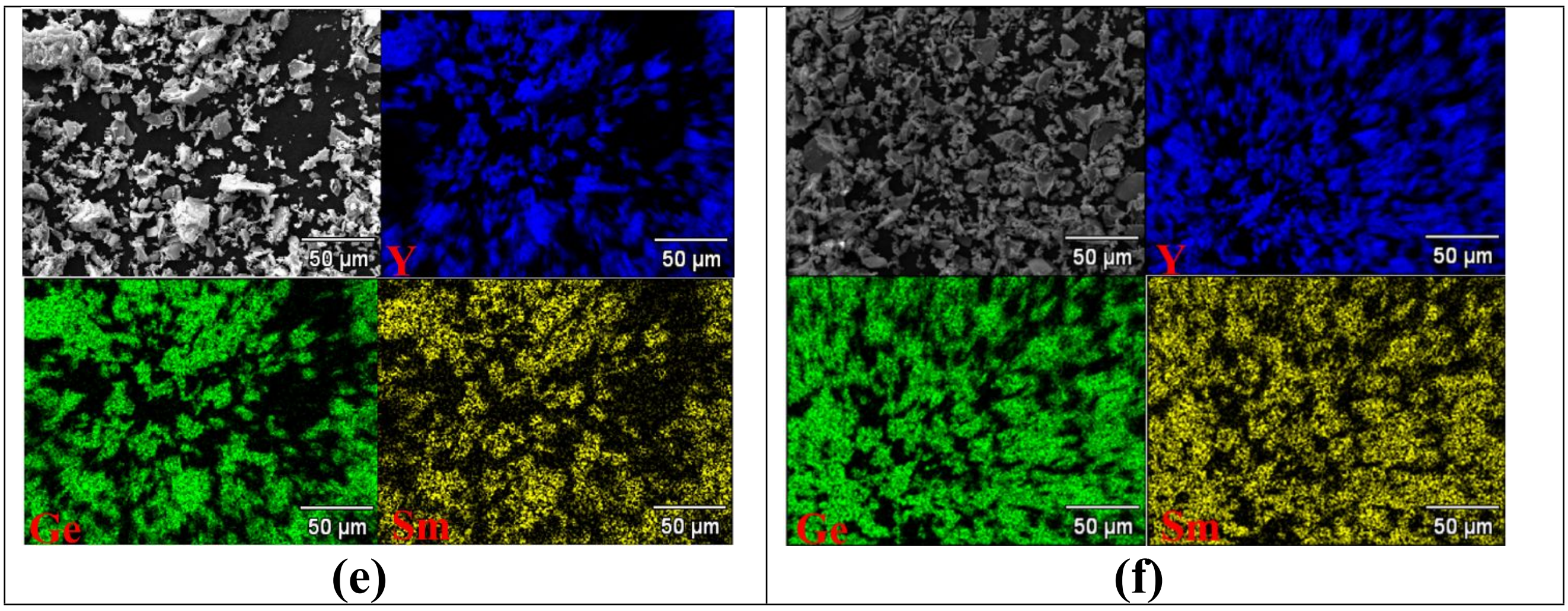

Figure S2. High resolution XPS spectra of Y3d, Ge2p, and O1s for $Y_2Ge_2O_7$:$x$$Sm^{3+}$ powders. (a), (b), and (c) for sample with 0.5 mol%, (d), (e), and (f) for sample with 2.0 mol%, and (g), (h), and (i) for sample with 4.0 mol%.

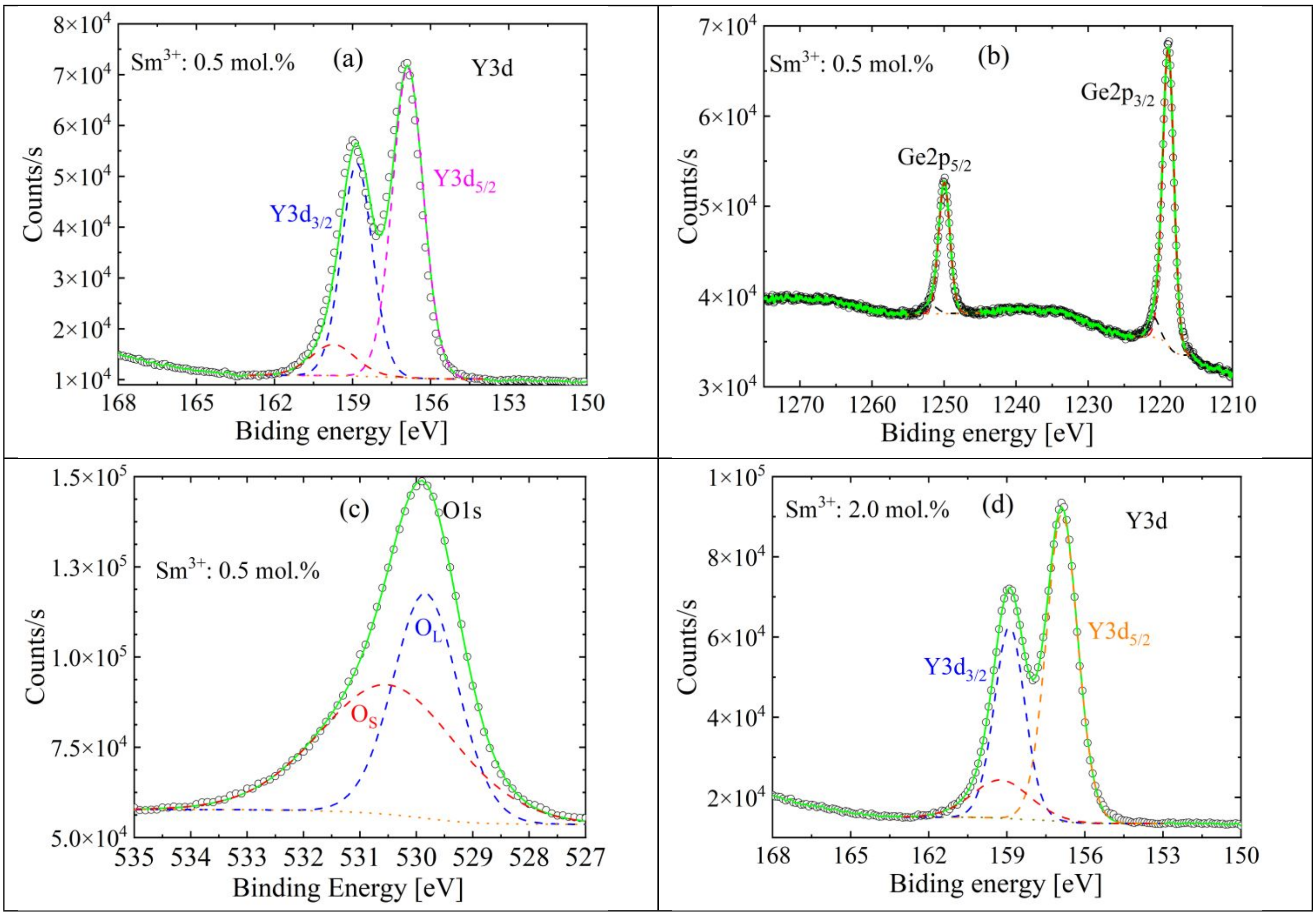

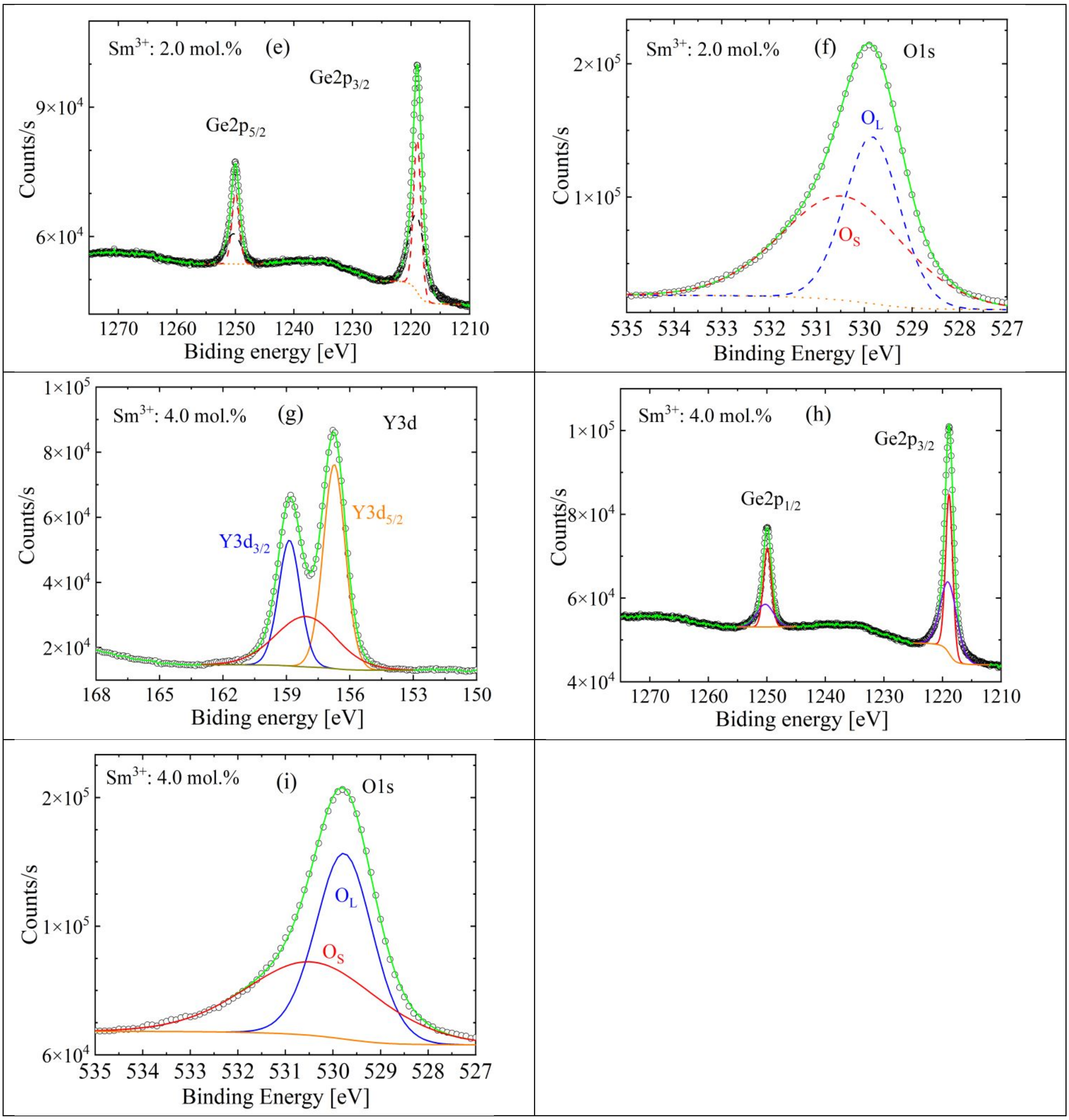
Sm3+: 2.0 mol.%
(e)
Ge2p3/2
Ge2p5/2
Counts/s
9×10^4
6×10^4
1270 1260 1250 1240 1230 1220 1210
Biding energy [eV]
Sm3+: 2.0 mol.%
(f)
O1s
OL
OS
Counts/s
2×10^5
1×10^5
535 534 533 532 531 530 529 528 527
Binding Energy [eV]
Sm3+: 4.0 mol.%
(g)
Y3d
Y3d5/2
Y3d3/2
Counts/s
1×10^5
8×10^4
6×10^4
4×10^4
2×10^4
168 165 162 159 156 153 150
Biding energy [eV]
Sm3+: 4.0 mol.%
(h)
Ge2p3/2
Ge2p1/2
Counts/s
1×10^5
8×10^4
6×10^4
4×10^4
1270 1260 1250 1240 1230 1220 1210
Biding energy [eV]
Sm3+: 4.0 mol.%
(i)
O1s
OL
OS
Counts/s
2×10^5
1×10^5
6×10^4
535 534 533 532 531 530 529 528 527
Binding Energy [eV]

Figure S3. Dependence of the integrated area of the Sm^{3+} $^4G_{5/2} \rightarrow {}^6H_{7/2}$ transition, red symbols, and **R/G** and **O/G** ratios, black and blue symbols, respectively with samarium concentration. The solid lines are guide for eyes.

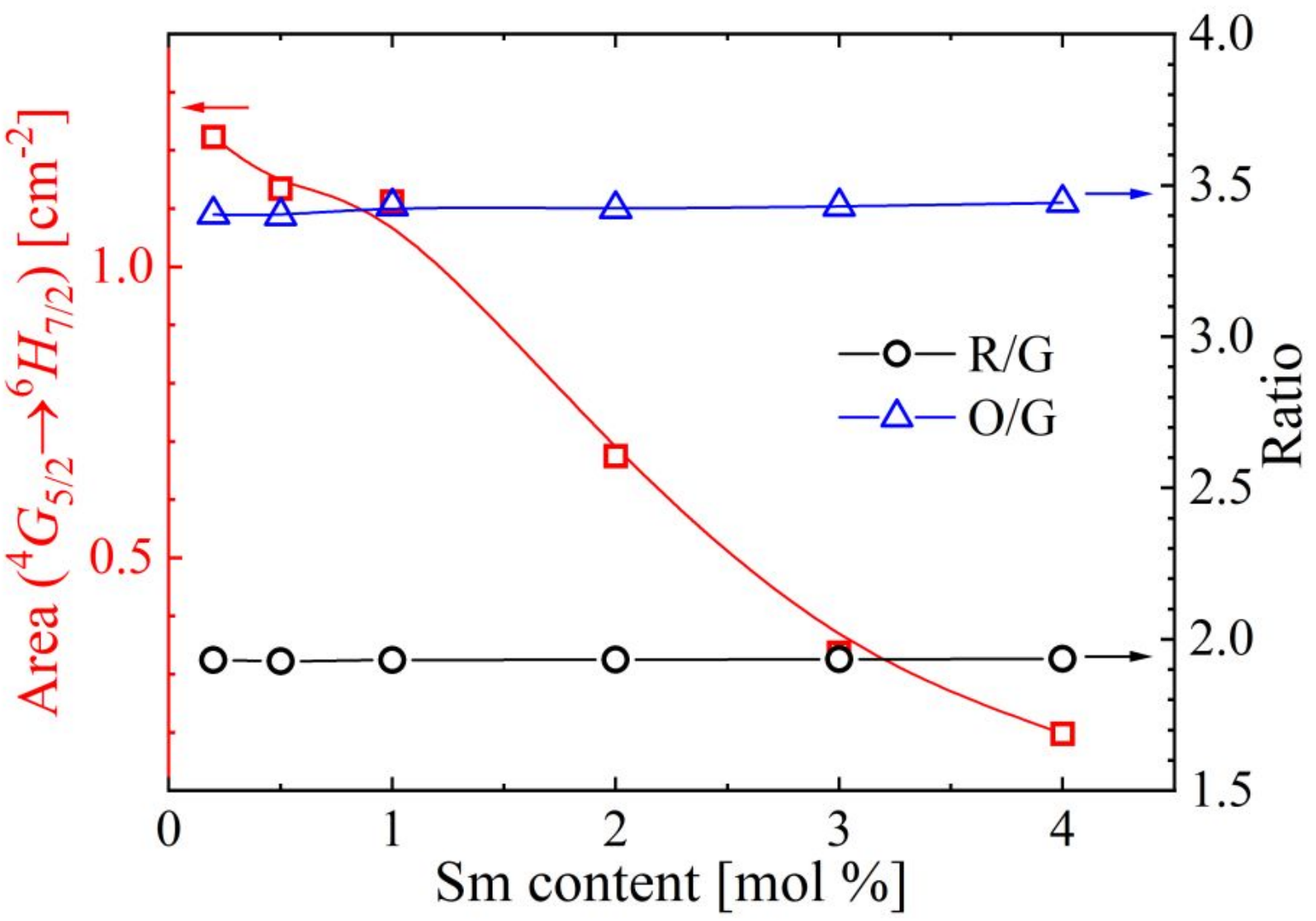

Figure S4. Calculated electronic band structure of the pristine $Y_2Ge_2O_7$ host matrix along high-symmetry directions in the Brillouin zone. The valence band maximum is set to 0 eV. The plot confirms a direct band gap, with both the valence band maximum and conduction band minimum located at the *Γ* point.

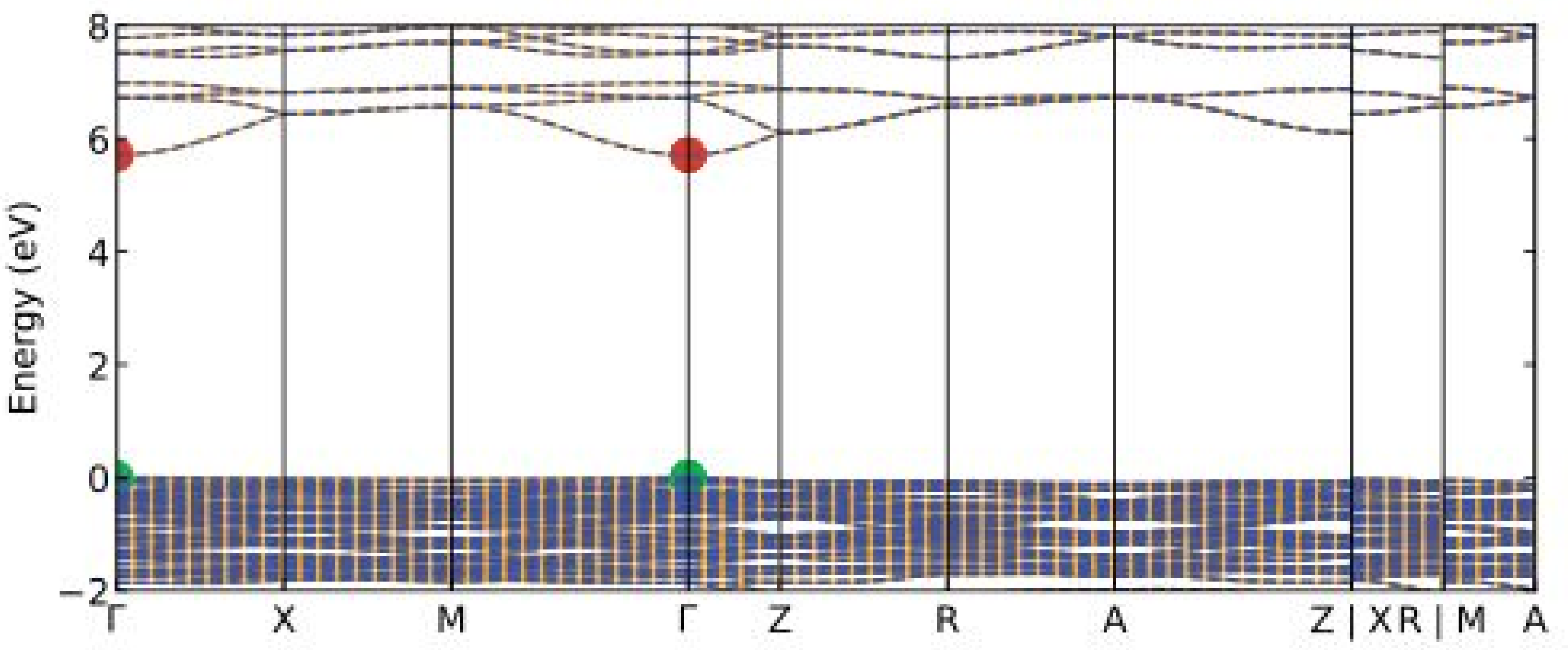

Figure S5. Total and partial density of states (DOS) for $Y_2Ge_2O_7$ doped with Sm^{3+}. (a) Single-dopant model ($x = 3.125$ mol%). Double-dopant model ($x = 6.250$ mol%) in (b) antiferromagnetic (AFM) and (c) ferromagnetic (FM) states. The Sm $4f$ levels (yellow) remain highly localized within the invariant band gap of the host matrix across all configurations. The Fermi level is set to zero.

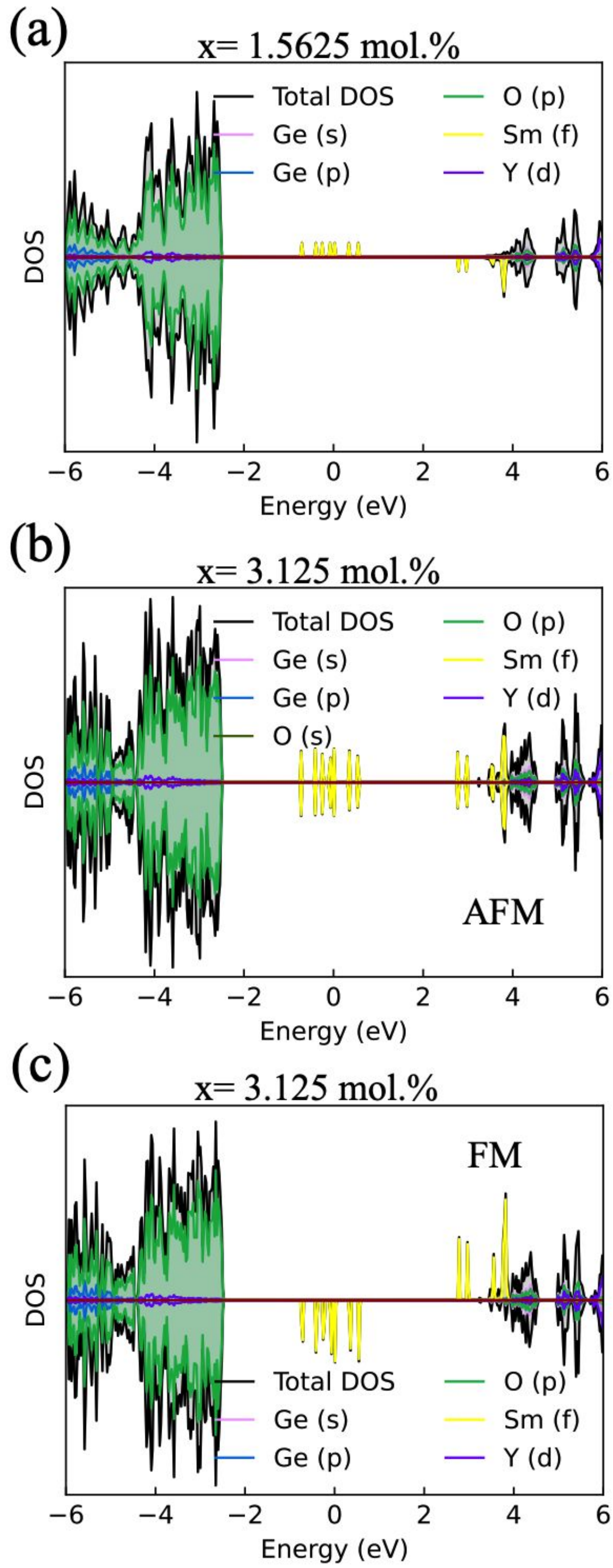

Table S2. External quantum yield (*eQY*) values of $Y_2Ge_2O_7$:$x$$RE^{3+}$ phosphors (RE = Sm^{3+}. Tb^{3+}. or Eu^{3+}).

***x* mol%**	***eQY* (%) – $Y_2Ge_2O_7$**		
	Sm^{3+}	Tb^{3+} [1]	Eu^{3+} [2]
0.2	0.52	0.05	0.12
0.5	0.73	0.4	0.01
1.0	0.50	0.5	0.28
2.0	0.36	1.0	0.52
3.0	0.18	--	1.18
4.0	0.10	1.2	0.60

Figure S6. Temperature dependence of the emission spectra for $Y_2Ge_2O_7$:$x$$Sm^{3+}$ in the 500 to 750 nm range under excitation at λ_{exc} = 402.7 nm.

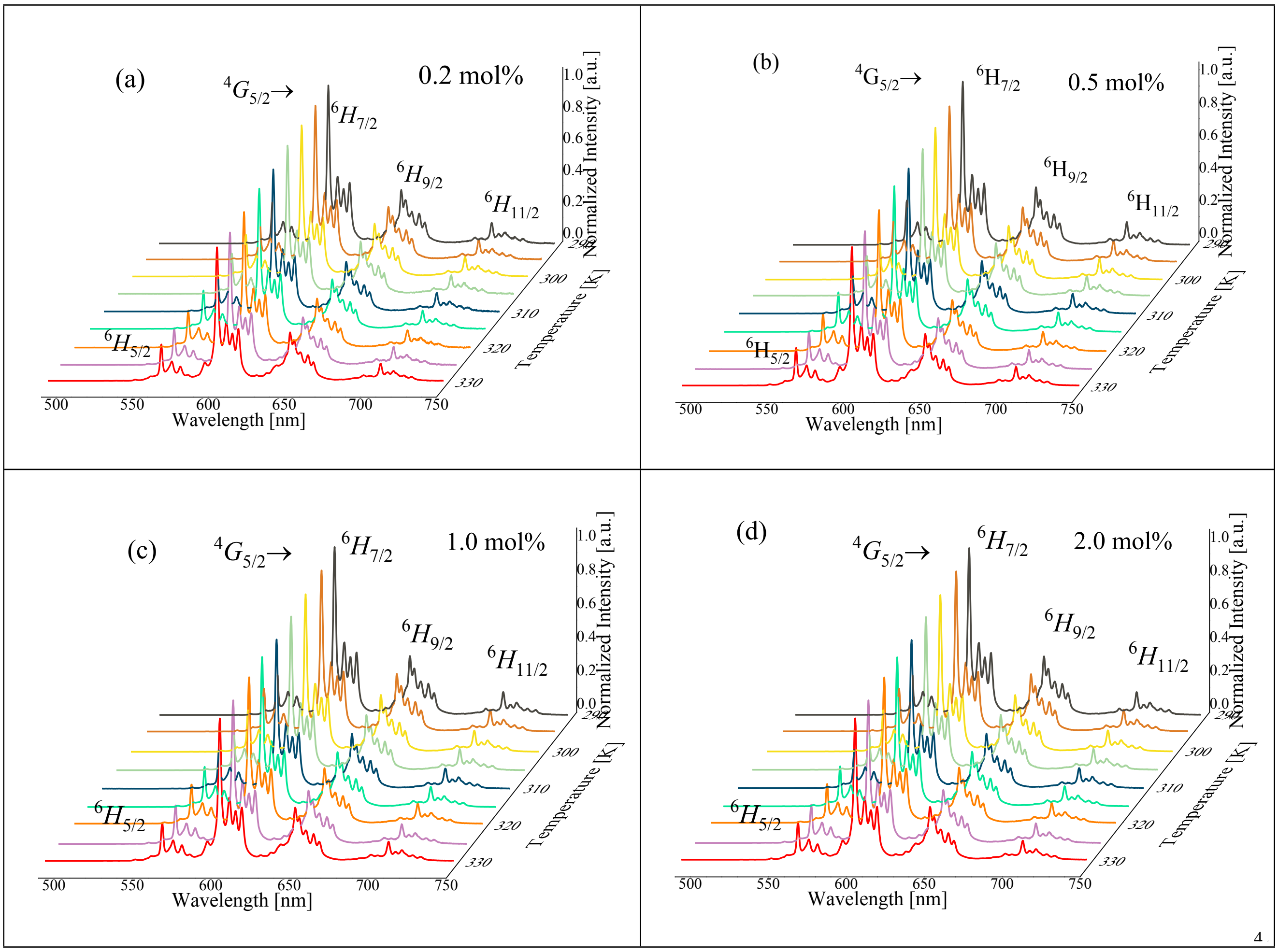

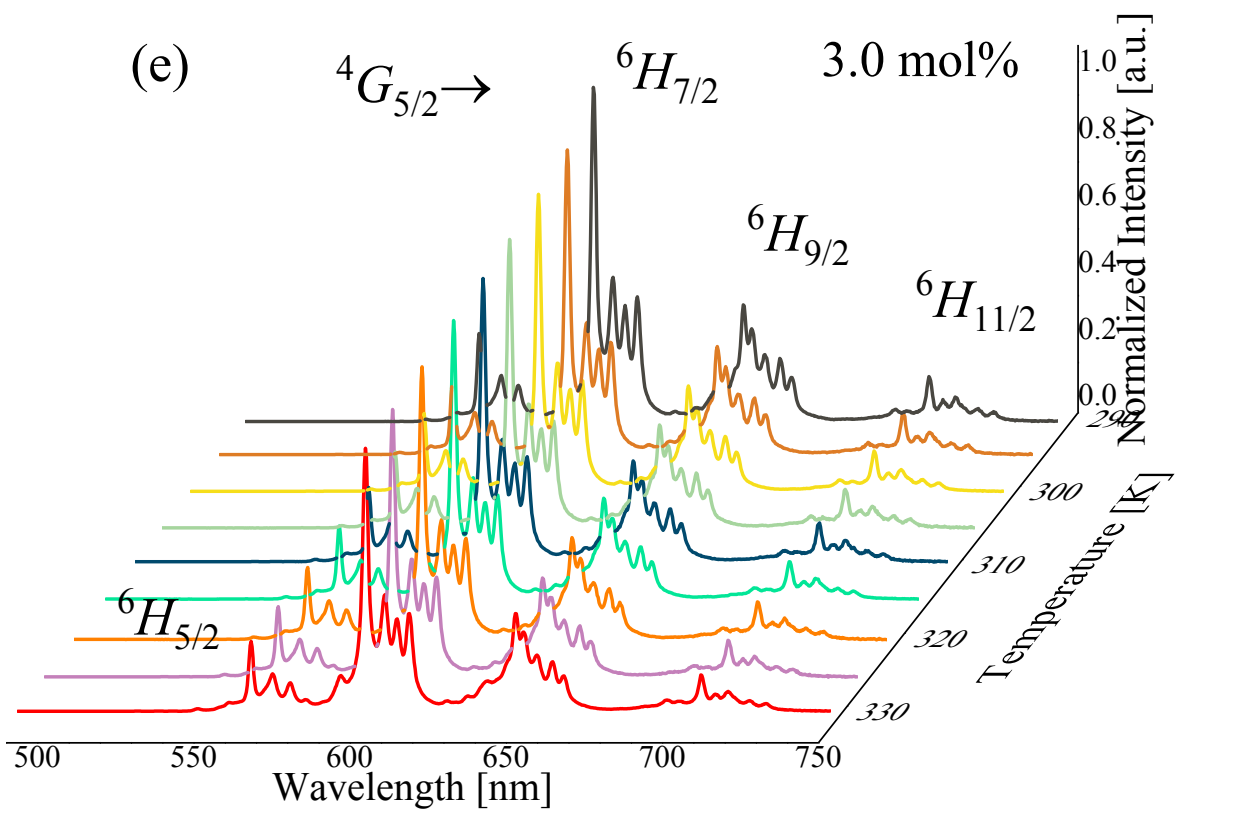

Figure S7. Temperature behaviour of the **R/G** (open symbols) and **O/G** (solid symbols) ratios for $Y_2Ge_2O_7$:$x$$Sm^{3+}$, under excitation at λ_{exc} = 402.7 nm.

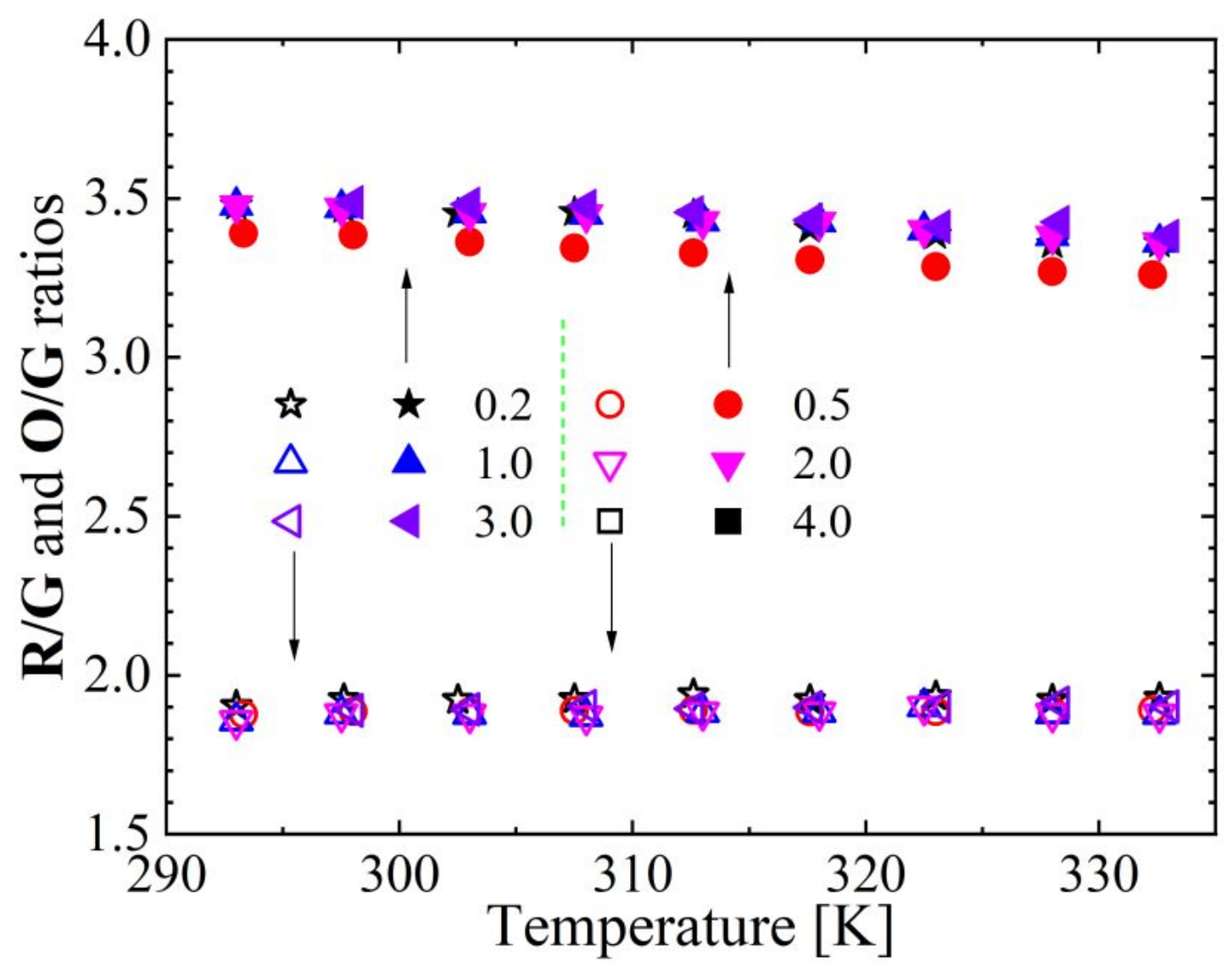

Figure S8. Emission spectrum of $Y_2Ge_2O_7:Sm^{3+}$ showing the selected integrated areas, I_1 = 563.8 – 565.5 nm, I_2 = 599.8 – 602.8 nm, I_3 = 648.4 – 650.8 nm and I_4 = 707.5 – 710.8 nm ranges, respectively for of ${}^4G_{5/2} \rightarrow {}^6H_{5/2}$, ${}^4G_{5/2} \rightarrow {}^6H_{7/2}$, ${}^4G_{5/2} \rightarrow {}^6H_{9/2}$, and ${}^4G_{5/2} \rightarrow {}^6H_{11/2}$ transitions.

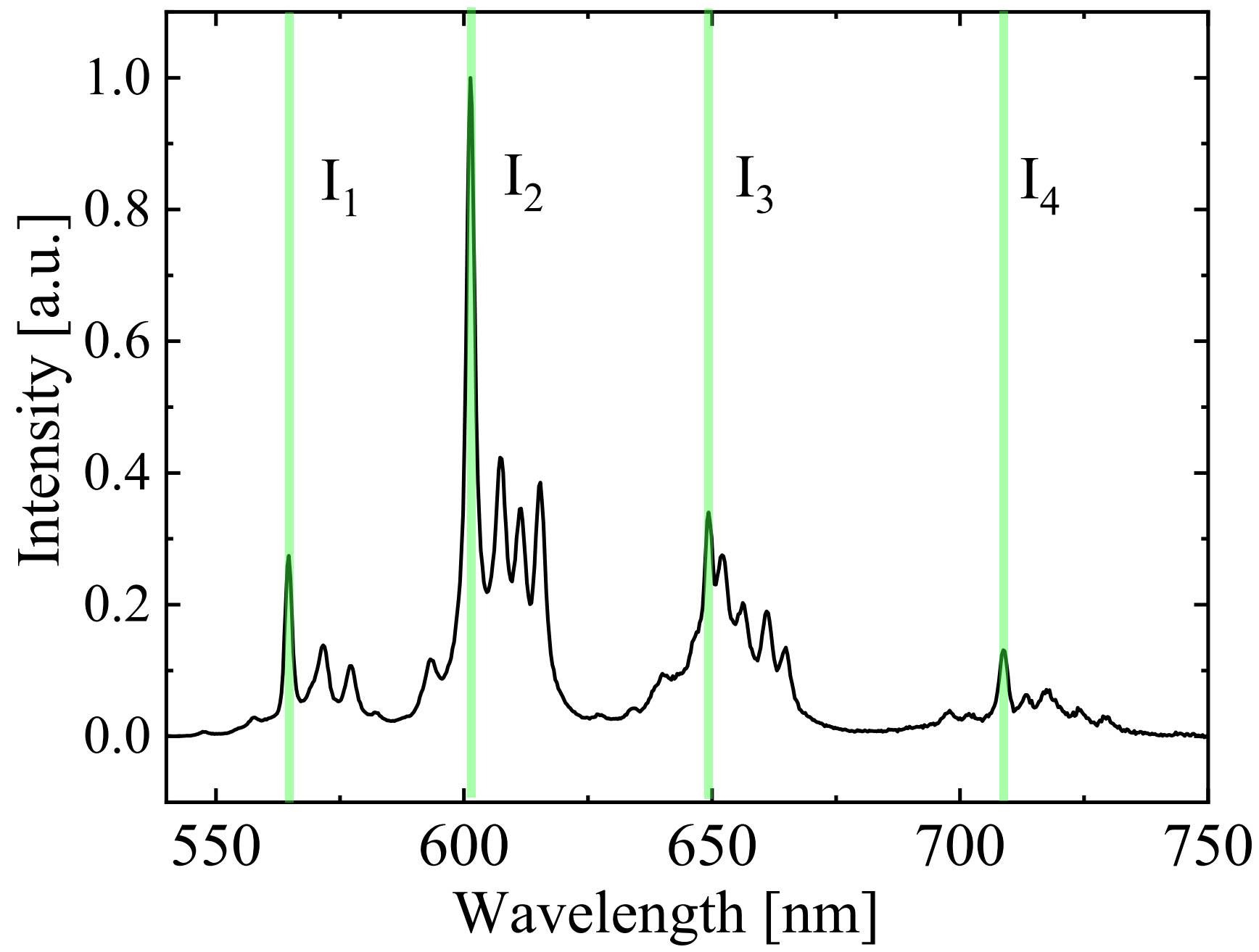

Table S3. CIE 1931 (*x.y*) colour coordinates of $Y_2Ge_2O_7:xSm^{3+}$ phosphors *vs* temperature.

0.2 mol %			0.5 mol %			1.0 mol %		
T [K]	*x*	*y*	*T [K]*	*x*	*y*	*T [K]*	*x*	*y*
293.2	0.615	0.385	293.3	0.614	0.385	293	0.615	0.385
297.6	0.615	0.385	298	0.614	0.385	297.5	0.614	0.385
302.5	0.614	0.385	303	0.614	0.385	303	0.614	0.385
307.5	0.614	0.385	307.5	0.614	0.386	308	0.614	0.385
312.6	0.614	0.385	312.6	0.614	0.386	313	0.614	0.386
317.6	0.614	0.386	317.6	0.613	0.386	318	0.614	0.385
323	0.613	0.386	323	0.613	0.386	322.5	0.614	0.386
328	0.613	0.386	328	0.613	0.386	328	0.614	0.386
332.3	0.613	0.386	332.6	0.613	0.387	332.6	0.613	0.386

2.0 mol %			3.0 mol %			4.0 mol %		
T [K]	*x*	*y*	*T [K]*	*x*	*y*	*T [K]*	*x*	*y*
293	0.615	0.384	293.5	0.618	0.381	293	0.615	0.385
298	0.615	0.385	298	0.618	0.382	298	0.614	0.385
303	0.614	0.385	303	0.617	0.382	303	0.614	0.385
308	0.614	0.385	308	0.617	0.382	308	0.614	0.386
313	0.614	0.385	312.6	0.617	0.382	313	0.614	0.386
318	0.614	0.386	317.7	0.616	0.383	318	0.613	0.386
323	0.613	0.386	323.2	0.616	0.383	323	0.613	0.386
328	0.613	0.386	328.3	0.615	0.384	328	0.613	0.386
333	0.613	0.386	333	0.614	0.385	333	0.613	0.387

Table S4. Correlated Colour Temperature (CCT) values of $Y_2Ge_2O_7$:$x$$Sm^{3+}$ phosphors *vs* temperature.

0.2 mol %		0.5 mol %		1.0 mol %	
T [K]	*CCT [K]*	*T [K]*	*CCT [K]*	*T [K]*	*CCT [K]*
293.2	1785.68	293.3	1782.86	293	1783.58
297.6	1784.84	298	1782.18	297.5	1783.11
302.5	1783.09	303	1779.54	303	1781.44
307.5	1782.49	307.5	1777.32	308	1780.2
312.6	1781.65	312.6	1775.69	313	1777.99
317.6	1776.79	317.6	1774.11	318	1778.01
323	1774.36	323	1771.23	322.5	1776.14
328	1771.63	328	1769.88	328	1776.14
332.3	1770.72	332.6	1768.88	332.6	1773.11

2.0 mol %		3.0 mol %		4.0 mol %	
T [K]	*CCT [K]*	*T [K]*	*CCT [K]*	*T [K]*	*CCT [K]*
293	1788.99	293.5	1819.42	293	1786.09
298	1785.2	298	1815.09	298	1781.45
303	1781.56	303	1812.13	303	1780.63
308	1780.06	308	1809.43	308	1777.75
313	1779.09	312.6	1805.6	313	1776.07
318	1776.04	317.7	1801.39	318	1774.58
323	1774.04	323.2	1785.47	323	1773.41
328	1772.31	328.3	1789.12	328	1772
333	1769.83	333	1784.15	333	1768.86

[1] D.E.T. dos Santos. A. Torquato. I.V. Barbosa. J.F. Carvalho. L.J.Q. Maia. R.C. de Santana. Temperature dependent photoluminescence properties of a Y2Ge2O7:Tb3+ phosphors. A dual band ratiometric luminescent thermometer. J. Alloys Compd. 1010 (2025) 177444. https://doi.org/10.1016/j.jallcom.2024.177444.

[2] I.V. Barbosa. L.J.Q. Maia. The influence of Eu3+ doping on the optical and structural properties of the Ge2Y2O7 crystalline phase through a soft chemical process. J Lumin 260 (2023) 119825. https://doi.org/10.1016/j.jlumin.2023.119825.
